# Gene Expression Data Mining Reveals the Involvement of GPR55 and Its Endogenous Ligands in Immune Response, Cancer, and Differentiation

**DOI:** 10.3390/ijms222413328

**Published:** 2021-12-11

**Authors:** Artur Wnorowski, Jakub Wójcik, Maciej Maj

**Affiliations:** Department of Biopharmacy, Medical University of Lublin, 20-059 Lublin, Poland; quba.wojcik@gmail.com (J.W.); maciej.maj@umlub.pl (M.M.)

**Keywords:** RNAseq data mining, putative cannabinoid receptor, lysophosphatidylinositol biosynthesis, peptide sensing, lysophosphatidylglucoside receptor, lysophospholipase bioactivity

## Abstract

G protein-coupled receptor 55 (GPR55) is a recently deorphanized lipid- and peptide-sensing receptor. Its lipidic endogenous agonists belong to lysoglycerophospholipids, with lysophosphatidylinositol (LPI) being the most studied. Peptide agonists derive from fragmentation of pituitary adenylate cyclase-activating polypeptide (PACAP). Although GPR55 and its ligands were implicated in several physiological and pathological conditions, their biological function remains unclear. Thus, the aim of the study was to conduct a large-scale re-analysis of publicly available gene expression datasets to identify physiological and pathological conditions affecting the expression of GPR55 and the production of its ligands. The study revealed that regulation of GPR55 occurs predominantly in the context of immune activation pointing towards the role of the receptor in response to pathogens and in immune cell lineage determination. Additionally, it was revealed that there is almost no overlap between the experimental conditions affecting the expression of GPR55 and those modulating agonist production. The capacity to synthesize LPI was enhanced in various types of tumors, indicating that cancer cells can hijack the motility-related activity of GPR55 to increase aggressiveness. Conditions favoring accumulation of PACAP-derived peptides were different than those for LPI and were mainly related to differentiation. This indicates a different function of the two agonist classes and possibly the existence of a signaling bias.

## 1. Introduction

G protein-coupled receptor 55 (GPR55) is a seven transmembrane receptor (7TM), initially recognized as a sensor for phytocannabinoids, pharmacologically active compounds of *Cannabis sativa* [1,2]. A principal psychoactive constituent of cannabis, Δ^9^-tetrahydrocannabinol (Δ^9^-THC), was identified as non-selective agonist of GPR55 [3], whereas a predominant non-psychotropic phytocannabinoid, cannabidiol (CBD), was established to block the receptor [4,5]. More recent efforts led to the identification of selective non-cannabinoid ligands of GPR55 [6,7], including several molecular species of endogenous phospholipids and peptides [8,9,10].

**Figure 1:**
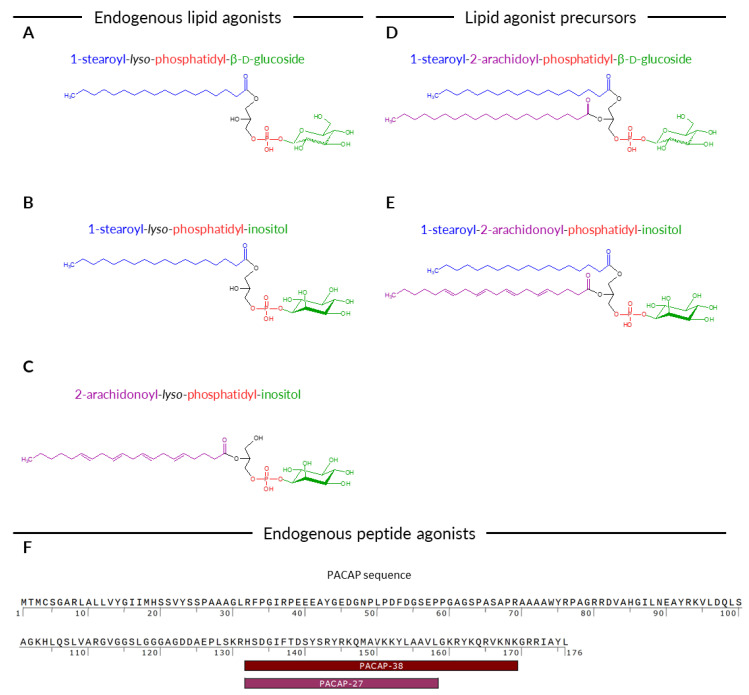
Structure of GPR55 ligands and their precursors. (**A**) 1-Stearoyl-*lyso*-phosphatidyl-β-d-glucoside (1-stearoyl-LysoPtdGlc or 1-stearoyl-LPGlc). (**B**) 1-Stearoyl-*lyso*-phosphatidylinositol (1-stearoyl-LPI). (**C**) 2-Arachidonoyl-*lyso*-phosphatidylinositol (2-arachidonoyl-LPI). (**D**) 1-Stearoyl-2-arachidoyl-phosphatidyl-β-d-glucoside (1-stearoyl-2-arachidoyl-PGlc). (**E**) 1-Stearoyl-2-arachidonoyl-phosphatidylinositol (1-stearoyl-2-arachidonoyl-PI). (**F**) Full-length pituitary adenylate cyclase-activating polypeptide (PACAP) with marked sequences of PACAP27 and PACAP38 peptides.

**Figure 2:**
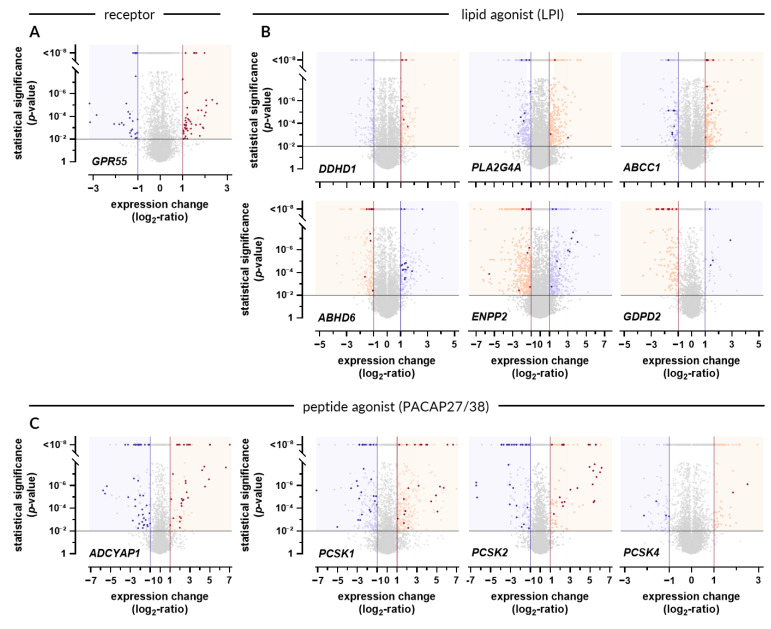
Expression profiles of *GPR55* and genes involved in the metabolism of LPI and PACAP27/38. (**A**) Volcano plot depicting experimental conditions (perturbations) leading to significant change in the expression of *GPR55*. Statistical significance threshold was set at *p*-value < 0.01 (black horizontal line). Conditions eliciting substantial upregulation were marked in dark red and were identified based on log_2_-ratio threshold of 1 (red vertical line). Conditions eliciting substantial downregulation of *GPR55* were marked in purple and were identified based on log_2_-ratio threshold of −1 (purple vertical line). All identified experimental conditions were listed in Supplementary Table S1 (upregulation) and Supplementary Table S2 (downregulation). (**B**) Perturbations favoring LPI accumulation (dark red dots) or LPI depletion (purple dots) were identified based on the expression profiles of genes involved in LPI production (*DDHD1*, *PLA2G4A*, and *ABCC1*) and in LPI degradation (*ABHD6*, *ENPP2*, and *GDPD2*). The identified experimental conditions were listed in Supplementary Table S3 (LPI accumulation) and Supplementary Table S4 (LPI depletion). (**C**) Perturbations favoring PACAP27/38 accumulation (dark red dots) or PACAP27/38 depletion (purple dots). The identified experimental conditions were listed in Supplementary Table S5 (PACAP27/38 accumulation) and Supplementary Table S6 (PACAP27/38 depletion). Pale red and pale purple dots indicate datapoints that crossed the *p*-value and log_2_-ratio thresholds but were excluded based on criteria described in the *Materials and Methods* section.

Phospholipids that activate GPR55 belong to lysoglycerophospholipids (LGPLs). The *lyso* prefix indicates that one of the two fatty acid chains was removed from the phospholipid molecule by hydrolysis. Thus, the hydrophobic tail of LGPLs contains only one esterified fatty acid chain to a glycerol core. Glycerol is further bound to a phosphate group linked to an alcohol or a carbohydrate (Figure 1A). Different species of LGPLs exist depending on the (I) type of acyl chain attached to the glycerol moiety, (II) attachment point of the acyl chain, and (III) nature of the chemical group linked to a phosphate moiety. Initial screens revealed that GPR55 is selective regarding the lipid structure, as only a limited number of endogenous phospholipids managed to activate the receptor [11]. The most potent endogenous GPR55 lipid identified so far is 1-stearoyl-*lyso*-phosphatidyl-β-d-glucoside (1-stearoyl-LysoPtdGlc or 1-stearoyl-LPGlc) [9]. It bears a stearic acid at *sn*-1 position and a single glucose moiety in the hydrophilic head of the molecule (Figure 1A). LGPL bearing an inositol moiety (i.e., lysophosphatidylinositol, LysoPtdIns, LPI) was also demonstrated to activate the receptor [11], although a preference appears to exist for gluco- over inositol-configured headgroups [12]. Among LPIs, 1-stearoyl-*lyso*-phosphatidylinositol (1-stearoyl-LPI; Figure 1B) and 2-arachidonoyl-*lyso*-phosphatidylinositol (2-arachidonoyl-LPI; Figure 1C) are capable of triggering the receptor, with the latter being more potent [8].

It is not clear how 1-stearoyl-LPGlc is synthesized in cells. Some preliminary studies indicated that its assembly depends on the supply of uridine diphosphate (UDP)-glucose, but the gene responsible for glucosylation of phosphatidic acid remains to be discovered [13]. Specific phospholipase catalyzing the cleavage of the arachidonoyl moiety from 1-stearoyl-2-arachidoyl-PGlc (Figure 1D) is also not known. On the contrary, key enzymes involved in the formation and degradation of LPIs are well established [14,15]. In general, LPIs are formed by hydrolysis of a single acyl chain from phosphatidylinositol (PI). Two GPR55 agonists, 1-stearoyl-LPI and 2-arachidonoyl-LPI, share the same precursor, i.e., 1-stearoyl-2-arachidonoyl-PI (Figure 1E), but are produced by distinct phospholipases. DDHD domain containing 1 phospholipase A_1_ (DDHD1 PLA_1_) cleaves the PI at the sn-1 position leading to the production of 2-arachidonoyl-LPI [16]. Cytosolic phospholipase A_2α_ (cPLA_2α_) encoded by *PLA2G4A* gene catalyzes hydrolysis of the sn-2 acyl chain of the PI precursor and causes the generation of 1-stearoyl-LPI [17,18]. LPIs produced by cytoplasmic phospholipases are expelled from the cells by the ATP-binding cassette transporter (ABCC1) [18]. Upon release to the extracellular space, LPI can bind to GPR55 and consequently activate signaling cascades downstream of the receptor [19].

Multiple enzymes catabolize LPI and contribute to the depletion of the GPR55 agonist pool [15]. LPIs can undergo complete diacylation leading to the formation of glycerophosphoinositol (GPI) and a fatty acid. This reaction is catalyzed by lysophospholipase A (lyso-PLA) encoded by *ABHD6* gene [20]. Alternatively, removal of the inositol moiety from LPI can occur, leading to the formation of lysophosphatidic acid (LPA). Lysophospholipase D (lyso-PLD) encoded by *ENPP2* gen and referred to as autotaxin drives the conversion of LPI into LPA [21,22]. Another route for LPI degradation involves the activity of lysophospholipase C (lyso-PLC) that cuts out the inositolphosphate from the sn-3 position generating a monoacylglycerol. Glycerophosphodiesterase 3 (GDE3, encoded by *GDPD2* gene) was recently identified as lyso-PLC digesting 2-arachidonoyl-LPI into 2-arachidonylglicerol (2-AG) and 1-stearoyl-LPI into 1-stearoylglicerol (1-SG) [23]. In contrast to LPIs, the metabolism of 1-stearoyl-LPGlc remains unexplored. However, some of the enzymes responsible for the processing of 1-stearoyl-LPI may also be involved in the synthesis and/or degradation of 1-stearoyl-LPGlc due to structural similarities between the compounds.

Despite being sensitive to lipids, GPR55 has evolutionary, sequence, and structural characteristics of peptide receptors [10]. Several endogenous peptides were discovered to activate GPR55 [10]. The most robust peptide agonist derives from pituitary adenylate cyclase-activating polypeptide (PACAP; *ADCYAP1* gene), residues 132–158, and is known as PACAP27 [24]. PACAP27 is active in receptor internalization and mass redistribution assays but not in β-arrestin recruitment, indicating that it may display signaling bias [25]. Apart from 27 amino acid peptide, longer cleavage variants (PACAP38 and a 45-amino acid variant) also display activity towards GPR55 [10]. Of note, PACAP27 is a well-established ligand for a cognate receptor, namely pituitary adenylate cyclase-activating polypeptide type I receptor, also known as PAC_1_ [26,27].

Full-length PACAP is processed by proprotein convertase (PC) 1 (*PCSK1* gene), PC2 (*PCSK2* gene), and PC4 (*PCSK3* gene) [28,29]. Upon consecutive cleavages catalyzed the convertases, peptides undergo α-amidation catalyzed by peptidylglycine alpha-amidating monooxygenase (PAM). Ultimately, C-terminally amidated mature PACAP38 and PACAP27 peptides are generated [27]. These peptides can be later degraded by dipeptidylpeptidase 4 (DPP4), a serine protease that cleaves dipeptides from the N-terminus of its substrates [30]. DPP4 is ubiquitously expressed membrane protein. The enzyme is shed from the plasma membrane as soluble circulating DPP4, whose activity can be readily detected in human serum [31]. Consequently, injected PACAP27 and PACAP38 are rapidly degraded with a half-life not exceeding 10 min [32,33].

PACAP27 exhibited the highest potency among known endogenous GPR55 ligands, yielding a half-maximal effective concentration (EC_50_) of 0.06 and 0.25 nM in dynamic mass redistribution and receptor internalization assay, respectively [10]. In the transforming growth factor-α (TGF-α) shedding assay, 1-stearoyl-LPGlc activated GPR55 with EC_50_ of 16 nM. In the same assay, inositol-bearing analogue yielded EC_50_ of 110 nM [9]. In an ERK activation study, the EC_50_ of 2-arachidonoyl-LPI was equal to 30 nM, whereas the value for 1-stearoyl-LPI was around 450 nM [11].

GPR55 is expressed in the brain, the gastrointestinal system, reproductive organs, lymphoid tissues, and blood cells. Overexpression in cancer cells has also been reported. Their ubiquitous presence suggests that GPR55 is involved in multiple biological actions. Until now, GPR55 signaling has been implicated in cancer aggressiveness [34], body weight regulation [35,36], induction of liver damage [23], inflammation [37], and neural development [9]. The aim of this study was to utilize gene expression data mining to identify new potential roles for this receptor and to cross-validate current findings on GPR55 function.

## 2. Results

### 2.1. GPR55 Upregulation Occurs Predominantly during Activation of Immune Cells

Intensified signaling downstream of GPR55 can occur in cells experiencing upregulation of the receptor or in cells exposed to increased agonist load. Vice versa, suppression of GPR55-dependent signaling is expected to happen upon receptor downregulation or under conditions favoring agonist depletion. Gene expression data mining was used to identify experimental conditions that match such profiles.

**Figure 3:**
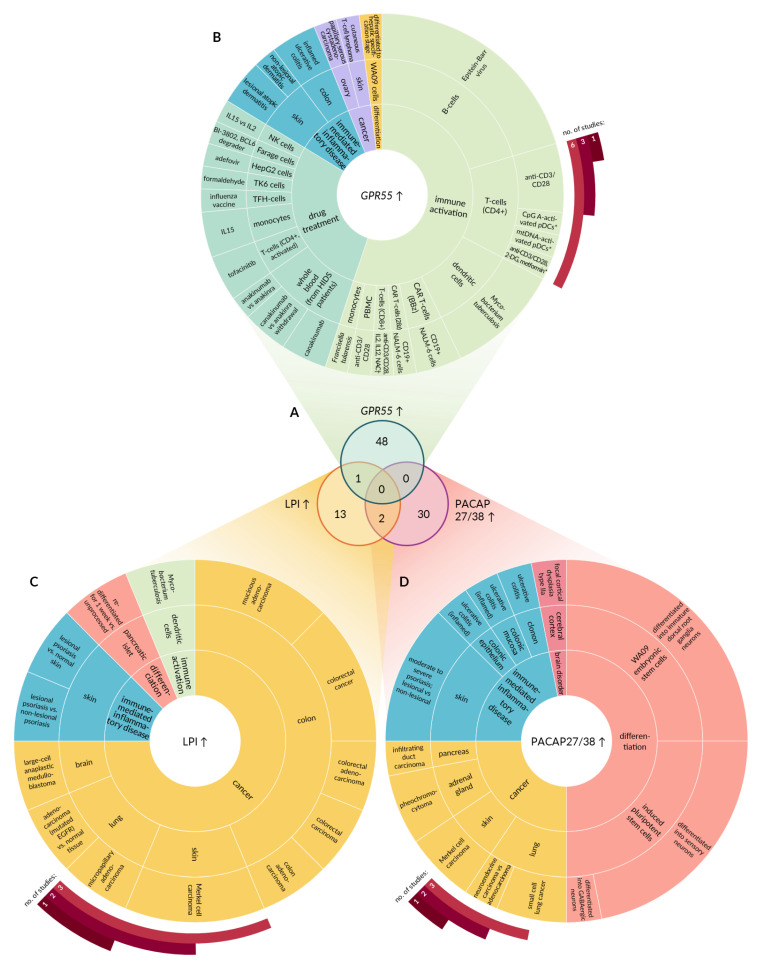
Perturbations leading to upregulation of GPR55 and its endogenous agonists. (**A**) Venn diagram demonstrating the number of experimental conditions characterized by increased GPR55 signaling capacity. Upper circle represents perturbations leading to significant GPR55 upregulation. Bottom circles represent conditions favoring either LPI (**left**) or PACAP27/38 (**right**) accumulation. (**B**) Ring chart depicting perturbations yielding increase in GPR55 expression. Inner ring represents class of the stimulus. Middle ring depicts tissue or cell type where the upregulation occurred. Outer ring provides the details on stimulus leading to GPR55 upregulation. Control conditions are omitted for vehicle, untreated, healthy, baseline, and normal controls. (**C**) Ring chart depicting perturbations favoring LPI accumulation. (**D**) Ring chart depicting perturbations favoring PACAP27/38 accumulation. BCL6, B-cell lymphoma 6 protein (zinc finger transcription factor); CAR-T, chimeric antigen receptor T cells; CpG A, type of short synthetic single-stranded DNA molecules containing unmethylated CpG dinucleotides; 2-DG, 2-deoxy-d-glucose; EGFR, epidermal growth factor receptor; HIDS, hyperimmunoglobulinemia D with recurrent fever; IL, interleukin; mtDNA, mitochondria DNA; NAC, N-acetyl-cysteine; PBMCs, peripheral blood mononuclear cells; PBMC, peripheral blood mononuclear cell; pDCs, plasmacytoid dendritic cells. *, vs. anti-CD3/28; †, vs. anti-CD3/28IL2, IL12 (without NAC).

**Figure 4:**
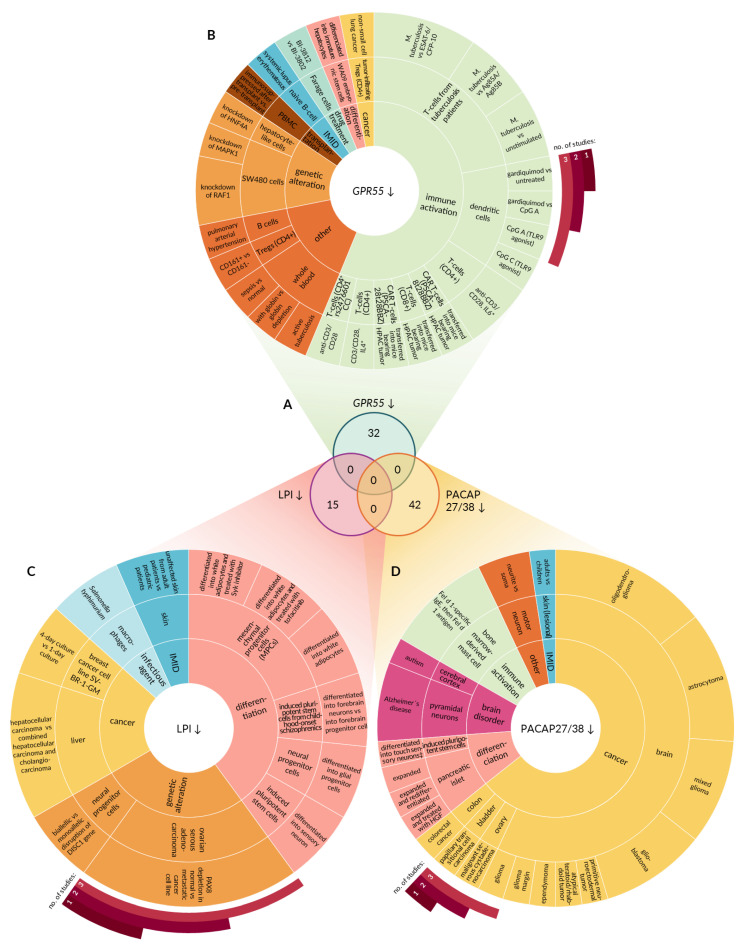
Perturbations leading to downregulation of GPR55 and its endogenous agonists. (**A**) Venn diagram demonstrating the number of experimental conditions characterized by decreased GPR55 signaling capacity. Upper circle represents perturbations leading to significant GPR55 downregulation. Bottom circles represent conditions favoring either LPI (**left**) or PACAP27/38 (**right**) depletion. (**B**) Ring chart depicting perturbations yielding decrease in GPR55 expression. Inner ring represents class of the stimulus. Middle ring depicts tissue or cell type where the downregulation occurred. Outer ring provides the details on stimulus leading to GPR55 downregulation. Control conditions are omitted for vehicle, untreated, healthy, baseline, and normal controls. (**C**) Ring chart depicting perturbations favoring LPI depletion. (**D**) Ring chart depicting perturbations that favor PACAP27/38 depletion. Ag85A, antigen of *Mycobacterium tuberculosis*; Ag85B, antigen of *M. tuberculosis*; CFP-10, antigen of *M. tuberculosis*; CpG A, type of short synthetic single-stranded DNA molecules containing unmethylated CpG dinucleotides; ESAT-6, antigen of *M. tuberculosis*; Farage, human non-Hodgkin’s B cell lymphoma line; gardiquimod, agonist of TLR7; HGF, hepatocyte growth factor; HNF4A, hepatocyte nuclear factor 4 alpha; HPAC, human pancreatic adenocarcinoma cell line; IMID, immune-mediated inflammatory disease; PBMC, peripheral blood mononuclear cell; pDCs, plasmacytoid dendritic cells; TLR7, Toll-like receptor 7; *, vs. anti-CD3/28; ‡, vs. into touch and cold sensory neurons.

First, transcriptomic datasets were queried for conditions leading to GPR55 upregulation (Figure 2A). A significant increase in *GPR55* mRNA was observed in only 49 experiments (Figure 3A; Supplementary Table S1). As depicted in Figure 3B, the identified experiments focused on immune activation (55%), response to drug treatment (29%), immune-mediated inflammatory diseases (IMIDs; 10%), cancer (4%), and differentiation (2%). *GPR55* upregulation was observed predominantly in the immune cells (67%) or in whole blood (10%). For instance, the expression of *GPR55* increased in primary resting B lymphocytes and in monocyte-derived dendritic cells upon infection with Epstein–Barr virus and live *Mycobacterium tuberculosis*, respectively. Elevation of *GPR55* level was also observed during activation of peripheral blood mononuclear cells, including CD4+ and CD8+ T-cells exposed to antibodies against CD3 and CD28. CD8+ T-cells engineered with a chimeric CD19 antigen receptor (CAR-T cells) displayed GPR55 upregulation when stimulated with leukemia cells expressing CD19. Drugs triggering GPR55 upregulation in the immune cells were identified, namely tofacitinib (immunosuppressant acting as janus kinase inhibitor), canakinumab (anti-inflammatory monoclonal antibody targeting interleukin-1β), and an influenza vaccine. Apart from immune cells, GPR55 upregulation was detected in established cell lines (8%), including WA09 embryonic stem cell line during late differentiation into hepatic specification stage, Farage lymphoma cell line exposed to BI-3802, a degrader of the transcription factor BCL6, HepG2 hepatocellular carcinoma cell line treated with adefovir (antiviral agent), and TK6 B-lymphoblastoid cell line subjected to formaldehyde. In addition, an increase in *GPR55* mRNA was observed in diseased skin (8%), colon (4%), and ovaries (2%), compared to normal tissues. For example, macroscopically inflamed sigmoid colon biopsies derived from patients diagnosed with ulcerative colitis were characterized by GPR55 upregulation in comparison to uninflamed hepatic flexure biopsies derived from affected patients and biopsies derived from healthy controls. A similar change in GPR55 expression was demonstrated in epidermal shaves of non-lesional skin of patients suffering from moderate to severe early-onset persistent atopic dermatitis versus healthy volunteers. GPR55 was detected in two types of cancers: papillary serous carcinoma and cutaneous T-cell lymphoma. The experimental conditions that led to the downregulation of GPR55 were identified based on gene expression data (Figure 2A). Significant suppression of GPR55 expression was observed in 32 studies (Figure 4A; Supplementary Table S2). Most of these studies focused on the activation of immune cells (56%; Figure 4B). The remaining experiments focused on the effects of genetic alterations (9%), differentiation (6%), transplantation (3%), IMIDs (3%), drug treatment (3%), cancer (3%), and other factors (16%). Downregulation of GPR55 occurred in blood cells (78%) or in established cell lines of different origin (19%). For instance, a decrease in *GPR55* mRNA was observed in dendritic cells exposed to Toll-like receptor 7 agonists, including gardiquimod and CpG oligodeoxynucleotides. CAR-T cells isolated 30 days after adoptive transfer into mice bearing HPAC-derived pancreatic tumor displayed a decrease in GPR55 in comparison to pre-infused cells. A similar trend was observed in SW480 colorectal adenocarcinoma cells upon knockdown of *MAPK1* (ERK2) or *RAF1* (c-RAF/Raf-1) genes, highlighting the involvement of mitogen-activated protein kinases (MAPKs) in the regulation of GPR55.

### 2.2. Conditions Promoting LPI Accumulation Exist in Tumors and LPI Suppression Is Preferred during Differentiation

Conditions affecting LPI levels were identified based on the expression profile of *DDHD1*, *PLA2G4A*, *ABCC1*, *ABHD6*, *ENPP2*, and *GDPD2* (Figure 2B). Pattern of gene expression supporting LPI accumulation was discovered in 16 experiments (Figure 3C and Supplementary Table S3). Apart from a single study on *M. tuberculosis*, there was no overlap with experiments characterized by GPR55 upregulation (Figure 3A). The environment supporting accumulation of both LPI and PACAP27/38 was present in primary and metastatic Merkel cell carcinoma biopsies in comparison to normal skin tissues (Figure 3C,D). Preference for LPI production over its degradation was predominant in tumors (75% of identified perturbations), including the neoplasms of colon/rectum, brain, skin, uterus, and lung (Figure 3C). Apart from that, a shift in gene expression favoring elevated LPI levels was observed in tissues affected by psoriasis (13%). A similar pattern of LPI-related gene expression occurred during re-differentiation of pancreatic islets (6%) and in response to *M. tuberculosis* infection (6%).

Conditions characterized by diminished supply and/or increased degradation of LPI were identified based on the expression profile of *DDHD1*, *PLA2G4A*, *ABCC1*, *ABHD6*, *ENPP2*, *GDPD2* (Figure 2B). The query on conditions characterized by diminished supply and/or increased degradation of LPI revealed 15 matching studies (Figure 4A and Supplementary Table S4). Of these, six focused on the differentiation process (40%; Figure 4C). The remaining studies investigated the effect of genetic alterations (27%), cancer (20%), infectious agents (7%), and IMIDs (7%). Putative LPI downregulation was observed during the generation of white adipocytes from mesenchymal progenitor cells (MPCs) and during the differentiation of neurons from induced pluripotent stem cells.

### 2.3. PACAP27 and PACAP38 Expression

The capacity for PACAP27/38 production was established based on expression profiles of PACAP polypeptide and enzymes involved in its processing (Figure 2C). The analysis revealed 32 perturbations favoring PACAP27/38 accumulation (Figure 3A and Supplementary Table S5). These perturbations relate to differentiation (50%), cancer (25%), IMIDs (22%), and brain disorders (3%; Figure 3D). For instance, increased PACAP27/38 production can be expected during differentiation of young sensory neurons from induced pluripotent stem cells (iPSC) and in immature dorsal root ganglia neurons (iDRGs) obtained by differentiation of WA09 embryonic stem cells. Conditions favoring PACAP27/38 production exist in lung, skin, adrenal, and pancreatic cancers (in comparison to normal tissues). A preference for PACAP27/38 upregulation was observed in intestinal epithelial cells isolated from inflammatory site of ascending colon of treatment-naïve pediatric patients with ulcerative colitis in comparison to control intestinal epithelial cells isolated from ascending colon of healthy children. A similar trend was present in lesional skin punch biopsies derived from patients with moderate-to-severe psoriasis compared to non-lesional and macroscopic normal skin punch biopsies derived from patients with moderate-to-severe psoriasis.

Downregulation of PACAP27/38 was observed in 42 studies (Figure 2C), predominantly related to cancer (64%; Figure 4D and Supplementary Table S6). Other conditions favoring PACAP27/38 depletion involved immune activation (10%), brain disorders (10%), differentiation (10%), and IMIDs (2%; Figure 4D). Most of the studies (31 out of 42) were performed using neuronal cells or tissues.

## 3. Discussion

Expression of GPR55 has been confirmed in several cell types and tissues. However, its physiological function has not yet been fully deciphered. Identification of experimental conditions characterized by altered expression of GPR55 and its endogenous agonists sheds some new light on the biological function of the receptor.

Here, we identified multiple perturbations related to immune activation that were associated with increased GPR55 expression. For instance, GPR55 upregulation occurred during the activation of naïve CD4 positive T-cells (helper cells). Generally, CD4+ naïve T-helpers undergo maturation in response to activation through antigen presenting cells (APCs) in order to polarize immune response, directing it towards certain defense mechanisms against particular type of infectious agent (e.g., a virus). Mature helper cells mediate their function mainly through differential paracrine secretion of cytokines. Cell activation and, in effect, maturation and differentiation show immense changes in the gene expression profile [38]. GPR55 expression was found to be increased during in vitro activation studies of helper cells. Activation was achieved with the aid of APCs or antibodies targeting the CD3 and CD28 receptors, which are crucial for cell activation during the antigen presentation process.

In a similar vein, activation of cytotoxic CAR-T cells upregulates GPR55 expression. CAR-T cells are engineered with chimeric artificial receptors for targeted immunotherapy in lymphomas. The chimeric artificial receptors are designed to facilitate activation with a particular antigenic target, which is usually CD19, expressed in many lymphoma cells. In in vitro studies involving CAR-T cells, it was shown that co-culture with a lymphoblastic leukemia cell line activated CAR-T cells, which responded with increased expression of GPR55.

Interestingly, in one of the studies on CD3/CD28-activated T helper cells, a treatment with a Janus kinase (JAK) inhibitor–tofacinib–further increased the expression of GPR55. This study was aimed at establishing the role of superenhancers in the regulation of the T-cell lineage. The JAK inhibitor preferentially affected the transcription of super-enhancer structured genes, which in T helper cells are mainly cytokine and cytokine receptor genes, which are the key determinants of the ‘identity’ of T cells. Thus, upregulation of GPR55 by tofacininb hints at the receptor role as a determinant of the T cell lineage.

On the other hand, some studies report downregulation of GPR55 in response to activation of CD4 cells, either with antibodies targeting the CD3 and CD28 receptors or in conjunction with interleukin 6 or interleukin 4 treatments [39,40,41]. Interleukin 6 can be described as a pro-inflammatory cytokine, but interleukin 4 is a polarization signal for helper cells to differentiate into Th2 subtype, further pointing to the determinant role of GPR55 in the lineage.

It is impossible to discern between cause and effect based on available data and deduce the exact role of differential GPR55 expression. Nonetheless, an association between GPR55 and immune processes is clearly evidenced by presented gene expression data. The connection is further supported by involvement of GPR55 in gastrointestinal inflammation [42,43]. Additionally, a recent report showed a direct chemotactic activity of 1-stearoyl-LPGlc on human monocytes [44]. These results are in congruence with previously reported data regarding 1-stearoyl-LPGlc acting as a GPR55 agonist in axonal guidance, where glia-derived 1-stearoyl-LPGlc regulated axon tract patterning [9]. This indicates the general role of GPR55 in directing cell motility. Other chemoattractant molecules such as chemokines have also been shown to act as differentiation mediators and thus as lineage determinants in T cells [45]. It is then tempting to speculate that GPR55 plays a role in both abovementioned scenarios, i.e., chemoattraction and differentiation of immune cells.

Human B lymphocytes infected with Epstein–Barr virus (EBV) exhibited increased expression of GPR55 compared to control B cells [46]. EBV is a type of human herpesvirus able to infect B-cell lymphocytes and epithelial cells. Infection might be either lytic or latent, which persists through the lifetime of a lymphocyte. EBV infection of B cells increases the expression of about 700 host genes, including noteworthy GPCRs previously assigned as EBV-induced genes 1 and 2, which were later renamed the CCR7 chemokine receptor and the GPR183 oxysterol receptor, the role of which is still not fully understood [47,48,49].

A study on monocyte-derived dendritic cells infected with Mycobacterium tuberculosis (MTB) has also shown an increase in GPR55 expression. MTB is a pathogenic, intracellular bacteria infecting primarily phagocytic immune cells. The literature data on the MTB influence on dendritic cells (DC) regarding changes in the host gene expression is inconclusive and contradictory [50]. Some studies report that MTB actively evades the immune response by impairing dendritic cells’ function, while other studies report that changes in activated dendritic cells are beneficial in fighting the MTB infection. Inconclusiveness is further perpetuated by another expression profiling study, which shows a decrease in GPR55 expression in the blood of patients with active tuberculosis [51]. Furthermore, a transcriptome profiling study conducted on active tuberculosis patients T cell activation also shows downregulation of GPR55. Isolated T cells were either infected with MTB, stimulated with only MTB antigens, or unstimulated. In this comparison, infected cells have shown downregulation of GPR55 in relation to the other groups [52]. It can be inferred that GPR55 can be clustered in a subgroup of genes, in which regulation is connected to the immune response to pathogens.

Atopic dermatitis (AD) is a chronic inflammation of the skin with unknown etiology. Pathophysiology involves the migration and activation of Th2 CD4+ helper T cells to the skin layers. These cells, when activated, release pro-inflammatory cytokines, including IL-4, IL-13, and IL-31 which led to the dermatitis. Our data-mining study revealed an increase in GPR55 expression in AD patients compared to healthy controls.

Psoriasis is another chronic IMID of the skin. It is characterized by cycles of sustained inflammation and remission driven by dysregulation in both innate and adaptive immunity. Here, we detected conditions that favor LPI and PACAP27/38 production in psoriatic lesions compared to nonlesional tissue.

Chronic inflammation is also a hallmark of another disease—ulcerative colitis (UC). It is characterized by inflammation of rectum and colon, sometimes spreading to systemic inflammation. The exact cause of the condition is unknown, but autoimmunity plays an important role, as evidenced by T-cell infiltration of the intestinal mucosa [53]. Here, we identified UC as a condition characterized by upregulation of GPR55 and increased PACAP27/38 production. Data from dextran sulfate sodium (DSS)-induced colitis model in mice indicate that the receptor contributes to intestinal inflammation, since GPR55-deficient animals develop significantly less severe colitis than wild types [54]. Yet, PACAP27/38 seem to have protective role in inflammatory bowel diseases, as PACAP-deficient mice exhibit more severe clinical symptoms of colitis in DSS model in comparison to normal controls [55]. PACAP upregulation was described previously in UC patients [56], and conditions favoring PACAP27/38 production are reported here. Agonist upregulation should mimic the effects of receptor overexpression. Yet, PACAP27/38 seem to have the opposite role to GPR55 in the context of UC. This may indicate that either PACAP-derived peptides ameliorate colitis in a GPR55-independent manner or show a signaling bias toward GPR55. The former may be linked to the well-established role of PACAP peptides as PAC_1_ ligands. The later effect may be related to the ability of PACAP27/38 to induce biased receptor activation, i.e., mass redistribution and receptor internalization without triggering β-arrestin signaling [10]. Thus, it is tempting to speculate that PACAP27/38 selectively induces receptor endocytosis to remove it from the cell surface and limits its exposure to other ligands that are capable to fully activate GPR55 and trigger downstream cell signaling related to inflammatory response. Interplay between GPR55 and PACAP27/38 in the context of inflammatory diseases required experimental investigation.

Although chemically different, both LPI and PACAP27/38 seem to function in an autocrine and paracrine manner, wherein they are synthetized and released from a cell and then bind to cell surface receptors on the same cell or nearby cells and alter their behavior. Unfortunately, no metabolomic data on LPI nor PACAP27/38 levels across a large spectrum of conditions exist. Additionally, no metabolic pathway reconstruction was carried out for the biosynthesis and degradation of these ligands, so it is currently not possible to calculate their production rates and concentrations under physiological and pathological conditions. Thus, we utilized gene expression data and simple selection criteria to identify conditions promoting either accumulation or depletion of LPI and PACAP27/38.

In general, conditions favoring LPI accumulation exist in cancer, while LPI downregulation is preferred during differentiation. In a simplistic view, carcinogenesis and differentiation are opposing processes; acquiring malignant traits by normal, differentiated cell increases its proliferative capacity, whereas normal cell differentiation from stem cells leads to reduction in cell division rate. GPR55 signaling has previously been associated with increased proliferation and motility of cancer cells, resulting in increased aggressiveness of tumors [34,57,58,59]. This analysis supports the concept that cancer-related GPR55 overactivation occurs predominantly due to the increased generation of endogenous agonist, and upregulation of GPR55 is secondary. However, direct measurements of LPI and LPGlc species in normal and tumor tissues are required to further corroborate the role of these endogenous lipids in cancer.

Studies on the involvement of PACAP27/38 into carcinogenesis and tumor progression have shown that the peptides may exert different effects depending on a model system. Genetic depletion of PACAP in mice increased medulloblastoma incidence, thereby demonstrating that PACAP exerts a potent inhibitory action on the induction and growth of these tumors [60]. The antiproliferative action of PACAP38 observed in primary medulloblastoma cell lines was PKA-dependent, indicating the involvement of Gα_s_-coupled PAC_1_ receptor [60]. The protective role of PACAP in the context of brain tumors was well depicted in this study. PACAP27/38 downregulation was predominant in the tumor tissues of the brain, highlighting importance of the peptides for tumor progression. In contrast to PKA-dependent antitumor action of PACAP peptides in the brain, their activity in the lung involves transactivation of the receptor tyrosine kinases through phospholipase C/Ca^2+^, consequently leading to increased cell proliferation [61,62,63]. Here, we detected that conditions favoring PACAP27/28 may exist in tumors of the lung, skin, adrenal gland, and pancreas, which, as supported by some of the literature data, could facilitate growth of cancer cells. The functional pleiotropism of PACAP27/38 could be attributed to splice variants of PAC_1_ receptor, which display differential coupling to Gα_q_/Ca^2+^ and Gα_s_/cAMP signaling [64]. As recently discovered, PACAP27/38 can also activate GPR55 [10]. Thus, it might be tempting to speculate that some of the activities of the peptides previously attributed to the interaction with PAC_1_ variants may arise from GPR55 binding. This hypothesis might be of particular interest, as GPR55 couples to Gα_q_ to increase intracellular calcium, similarly to PAC_1_ [3].

The limitation of this data mining study relates to reporting bias and database bias. One has to keep in mind that we were not able to identify conditions that were never studied using RNAseq or microarrays techniques. Additionally, only around 66% of experiments deposited in the Genevestigator database were carried out using arrays without any probe for GPR55. It is important to consider that a large number of hits in a certain research area may be related to multiple similar studies related to the same phenomenon, which may give a biased impression of a significance.

## 4. Materials and Methods

### 4.1. Software

Gene expression data mining was conducted using Genevestigator 8.0.2 (Nebion AG, Zurich, Switzerland) [65]. The data sets considered for the analysis were restricted to human samples. Gene expression values were expressed as log_2_ of (Transcripts Per Million (TPM) + 1). Scatter dot plots were created using Prism v8.4.3 (GraphPad Software Inc, San Diego, CA, USA), and ring charts were drawn in Microsoft Excel v2102 (Microsoft Corporation, Redmond, California, USA).

### 4.2. Perturbations

**Figure 5:**
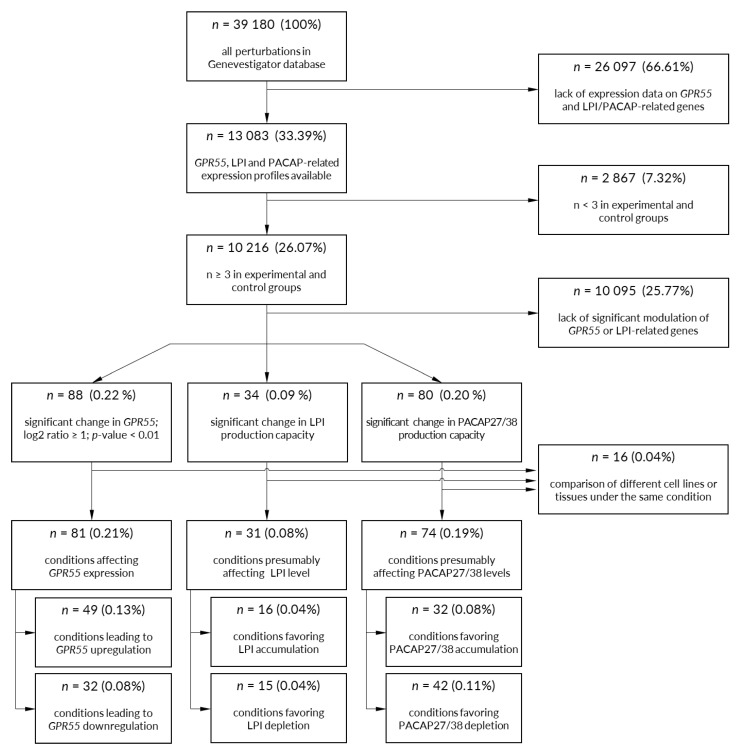
Pipeline for selection of conditions significantly affecting the expression of GPR55 and its endogenous agonists. Open gene expression datasets were used to identify experimental conditions affecting the expression of GPR55 and the production capacity of its endogenous agonists—LPI and PACAP27/38. See *Materials and Methods* for details regarding selection criteria.

Experimental conditions (e.g., chemicals, diseases, hormones, stresses, mutations) affecting the expression of *GPR55* and other genes of interest were identified using *Perturbation* tool from Genevestigator suit. The data originated from the RNAseq experiments and the following microarrays: Affymetrix (HT HG-U133+ PM, Human Exon 1.0 ST, Human Gene 1.0 ST, Human Gene 1.1 ST, Human Gene 2.0 ST, Human Gene 2.1 ST, Human Genome U133 Plus 2.0, Human Genome U219, Human Transcriptome Array 2.0) and Illumina (Human Whole Genome DASL HT-12 V4.0, HumanHT-12 V3.0, HumanHT-12 V4.0). The microarray platforms were selected on the basis of the availability of probes corresponding to all genes of interest. Experiments carried out with a low number of samples per group (*n* < 3) were excluded from the analysis. For each gene of interest within every study, the log_2_ ratio was calculated as the difference between the mean log_2_ expression for experimental samples and the mean log_2_ expression for corresponding control samples from the same experiment. The *p*-values were calculated by the Genevestigator. For microarray data, the *p*-values were computed using the t-test. For RNA sequencing data, the t-test was adjusted to take into account the nature of the underlying read mapping data. An experimental condition was considered to significantly affect the expression of the gene of interest when the *p*-value < 0.01, and log_2_ ratio ≥1 (upregulation) or ≤−1 (downregulation). Studies comparing expression patterns between two different tissues or cell lines under the same conditions were excluded. The number of experimental conditions retained for further analysis after the application of each constrain is depicted in Figure 5.

### 4.3. Assessment of LPI Production Capacity

No large-scale lipidomic data on LPI levels exist. Thus, expression of genes involved in LPI metabolism was used a surrogate marker for LPI production capacity. It was assumed that a synchronous change in the expression of genes directly involved in LPI metabolism (listed in Table 1) would substantially alter LPI levels. Based on this assumption, each experiment was scored according to the following criteria:

- The *LPI-score* was increased by one point (+1) whenever a gene positively affecting LPI level was upregulated or a gene negatively affecting LPI level was downregulated;
- Conversely, the *LPI-score* was decreased by on point (−1) whenever a gene positively affecting LPI level was downregulated or a gene negatively affecting LPI level was upregulated.

Experimental conditions yielding the *LPI-score* of 3 or more were considered to favor LPI accumulation. If the *LPI-score* was equal or lower than −3, the conditions were described as promoting LPI depletion.

### 4.4. Assessment of PACAP27 and PACAP38 Production Capacity

Capacity to accumulate PACAP27 and PACAP38 was estimated based on the expression profile of genes involved in the metabolism of the peptides (Table 2). It was assumed that efficient peptide generation requires the presence of precursor polypeptide (*ADCYAP1* gene), polypeptide processing enzyme (*PCSK1*, *PCSK2*, and *PCSK4* genes), and limited supply of peptide degrader (*DPP4* gene). Expression of processing enzymes was scored as described below:

- The *PC-score* was increased by one point (+1) whenever a convertase-coding gene (*PCSK1*, *PCSK2*, or *PCSK4*) was upregulated;
- The *PC-score* was decreased by one point (−1) whenever a convertase-coding gene (*PCSK1*, *PCSK2*, or *PCSK4*) was downregulated.

Based on the assumptions described above, conditions favoring PACAP27/38 accumulation were deemed to occur when all following conditions were met: (I) upregulation of *ADCYAP1* gene; (II) *PC-score* ≥ 1; (III) no upregulation in *DPP4* gene.

Vice versa, a decrease in PACAP27/38 load was expected to occur in experiments characterized by a simultaneous downregulation of *ADCYAP1*, drop in *PC-score* to −1 or less, and lack of *DPP4* upregulation.

## 5. Conclusions

Expression of GPR55 and its endogenous agonists is controlled independently. GPR55 regulation occurs predominantly in immune cells, where the receptor acts as an immune mediator and chemotaxis driver. The accumulation of the lipid agonist LPI occurs in cancer cells, indicating that tumors may hijack the natural motility-related function of GPR55 to increase invasiveness. On the contrary, peptide agonist PACAP27/38 is downregulated in cancer, which may be indicative for lack of its specificity as GPR55 ligand (PACAP27/38 activates also PAC_1_ receptor) or signaling bias at GPR55.

## Figures and Tables

**Table 1:** Genes modulating LPI levels.

Gene Symbol	Full Name (Gene; Protein)	Effect on LPI	Mode of Action	Ref.
*DDHD1*	DDHD domain containing 1; phospholipase DDHD1	positive	involved in the formation of 2-arachidonoyl-LPI (endogenous agonist)	[15,16]
*PLA2G4A*	phospholipase A2 group IVA; cytosolic phospholipase A2	positive	involved in the formation of 2-stearotyl-LPI (endogenous agonist)	[15,18,19]
*ABCC1* (*MRP1*)	ATP binding cassette subfamily C member 1; multidrug resistance-associated protein 1	positive	pumps LPI out of the cell enabling autocrine and paracrine activation of GPR55	[18,19]
*ABHD6*	abhydrolase domain containing 6, acylglycerol lipase; monoacylglycerol lipase ABHD6	negative	has lysophospholipase A activity; degrades LPI into GPI and a fatty acid, depleting agonist pool	[20]
*ENPP2* (*ATX*)	ectonucleotide pyrophosphatase/phosphodiesterase 2; autotaxin	negative	has lysophospholipase D activity; degrades LPI into LPA	[21,22]
*GDPD2* (*GDE3*)	glycerophosphodiester phosphodiesterase domain containing 2; glycerophosphoinositol inositolphosphodiesterase GDPD2, glycerophosphodiesterase 3	negative	has lysophospholipase C activity; degrades LPI into 2-AG; generates CB2 agonist from LPI	[23]

Gene symbols and names are from HUGO Gene Nomenclature Committee (genenames.org; access date: 09.12.2021). Common aliases for gene symbols are provided in brackets. Protein names are from UniProt database (uniprot.org; access date: 09.12.2021) and literature.

**Table 2:** Genes modulating PACAP27 and PACPA38 levels.

Gene Symbol	Full Name (Gene; Protein)	Effect on PACAP27/38	Mode of Action	Ref.
*ADCYAP1* (*PACAP*)	adenylate cyclase activating polypeptide 1; pituitary adenylate cyclase-activating polypeptide	positive	undergoes cleavage that generates PACAP-27 peptide (endogenous agonist of GPR55)	[10]
*PCSK1* (*PC1*)	proprotein convertase subtilisin/kexin type 1; neuroendocrine convertase 1; proprotein convertase 1	positive	cleaves ADCYAP1-encoded polypeptide into shorter PACAP-27	[27,28]
*PCSK2* (*PC2*)	proprotein convertase subtilisin/kexin type 2; neuroendocrine convertase 2; proprotein convertase 2	positive	cleaves ADCYAP1-encoded polypeptide into shorter PACAP-27	[27,28]
*PCSK4* (*PC4*)	proprotein convertase subtilisin/kexin type 4; proprotein convertase 4	positive	cleaves ADCYAP1-encoded polypeptide into shorter PACAP-27	[27,29]
*DPP4* (*CD26*, *ADCP2*)	dipeptidyl peptidase 4; cluster of differentiation 26, adenosine deaminase complexing protein 2	negative	exopeptidase with a dipeptidyl peptidase activity; degrades PACAP-27	[27]

Gene symbols and names are from HUGO Gene Nomenclature Committee (genenames.org; access date: 09.12.2021). Common aliases for gene symbols are provided in brackets. Protein names are from UniProt database (uniprot.org; access date: 09.12.2021) and literature.

## Data Availability

Raw data analyzed in this study are available through Genevestigator software that can be obtained at genevestigator.com.

## Supplementary Materials

The following are available online at https://www.mdpi.com/article/10.3390/ijms222413328/s1.
