# Supplementary material for: Gene Expression Data Mining Reveals the Involvement of GPR55 and Its Endogenous Ligands in Immune Response, Cancer, and Differentiation
Source: Int J Mol Sci. 2021 Dec 11;22(24):13328. doi: 10.3390/ijms222413328 (PMC8707311; doi:10.3390/ijms222413328)
Supplement: Supplementary file 1 [file ijms-22-13328-s001.zip › ijms-1485450-supplementary.pdf]

Gene expression data mining reveals the involvement of GPR55 and its endogenous ligands in immune response, cancer, and differentiation

Supplemetary Materials

Authors: Artur Wnorowski, Jakub Wójcik, Maciej Maj
Correspondence: artur.wnorowski@umlub.pl

Table of contents

page 2	Supplementary Table S1	Experimental conditions leading to GPR55 upregulation
page 8	Supplementary Table S2	Experimental conditions leading to GPR55 downregulation
page 13	Supplementary Table S3	Experimental conditions promoting LPI accumulation
page 14	Supplementary Table S4	Experimental conditions promoting LPI depletion
page 17	Supplementary Table S5	Experimental conditions promoting PACAP27/38 accumulation
page 20	Supplementary Table S6	Experimental conditions promoting PACAP27/38 depletion

Supplementary Table S1.

Experimental conditions leading to *GPR55* upregulation.

		Experimental group				Control group			
ID	Repository	Stimulus	Description	Anatomical Part or Cell line or Neoplasm	n	Stimulus	Description	Anatomical Part or Cell line or Neoplasm	n
1	GSE125974	Epstein-Barr virus study 1 (28d)	Primary human resting B lymphocytes infected with B95.8 Epstein-Bar virus for 28 days.	B-cell	3	Epstein-Barr virus study 1 (0d)	Primary human resting B lymphocytes infected with B95.8 Epstein-Bar virus for 0 day.	B-cell	3
2	GSE60482	tofacitinib study 1 (0.3uM)	Tofacinitib (0.3uM) treated in vitro activated T-cells obtained from blood of a healthy donor. Naïve CD4+CD45RA+CD45RO− T cell population was FACS-sorted from peripheral blood mononuclear cells isolated by Ficoll paque. T-cells were activated by plate-bound anti-CD3/anti-CD28 in supplemented RPMI 1640 medium containing 10% FCS for 3 days and cultured in the presence of IL-2 for 1 day. During T cell activation, cells were treated with 0.3uM tofacitinib, a Janus kinase inhibitor.	peripheral blood CD4 activated T-cell (unspecified)	4	untreated activated T-cell sample	In vitro activated T-cells obtained from blood of a healthy donor. Naïve CD4+CD45RA+CD45RO− T cell population was FACS-sorted from peripheral blood mononuclear cells isolated by Ficoll paque. T-cells were activated by plate-bound anti-CD3/anti-CD28 in supplemented RPMI 1640 medium containing 10% FCS for 3 days and cultured in the presence of IL-2 for 1 day.	peripheral blood CD4 activated T-cell (unspecified)	4
3	GSE125974	Epstein-Barr virus study 1 (14d)	Primary human resting B lymphocytes infected with B95.8 Epstein-Bar virus for 14 days.	B-cell	3	Epstein-Barr virus study 1 (0d)	Primary human resting B lymphocytes infected with B95.8 Epstein-Bar virus for 0 day.	B-cell	3
4	GSE125974	Epstein-Barr virus study 1 (21d)	Primary human resting B lymphocytes infected with B95.8 Epstein-Bar virus for 21 days.	B-cell	3	Epstein-Barr virus study 1 (0d)	Primary human resting B lymphocytes infected with B95.8 Epstein-Bar virus for 0 day.	B-cell	3
5	GSE125974	Epstein-Barr virus study 1 (7d)	Primary human resting B lymphocytes infected with B95.8 Epstein-Bar virus for 21 days.	B-cell	3	Epstein-Barr virus study 1 (0d)	Primary human resting B lymphocytes infected with B95.8 Epstein-Bar virus for 0 day.	B-cell	3
6	GSE109843	T cell activation study 12 (pDC-mtDNA; 6d)	Naive CD4 T cells activated by co-culture with mitochondrial DNA (mtDNA)-activated plasmacytoid dendritic cells (pDC). pDCs were FACS-sorted from healthy blood as Lineage (CD3, CD14, CD16, CD19, CD20, CD56)- HLA-DR+ CD11c- CD123+ cells and activated with 40% (vol/vol) oxidized mtDNA-containing neutrophil supernatant for 24 h (healthy neutrophils were pre-incubated with interferon α2β and treated with anti-RNB IgG from SLE sera). Naive CD4 T cells were isolated from healthy blood as negative for CD8, CD14, CD16, CD19, CD20, CD25, CD36, CD56, CD61, CD66b, CD123, HLA-DR, TCRγ/δ, glycophorin A and CD45RO, labeled with carboxyfluorescein succinimidyl ester (CFSE) and co-cultured with mtDNA-activated pDCs (pDC:T cell ratio 1:6) for 6 days. For analysis, primed T cells were sorted from co-culture as CFSElow and CD123- cells.	peripheral blood CD4 naive T-cell	3	T cell activation study 12 (CD3/CD28; 6d)	Naive CD4 T cells activated by co-culture with anti-CD3/CD28-coated beads. Naive CD4 T cells were isolated from healthy blood as negative for CD8, CD14, CD16, CD19, CD20, CD25, CD36, CD56, CD61, CD66b, CD123, HLA-DR, TCRγ/δ, glycophorin A and CD45RO, labeled with carboxyfluorescein succinimidyl ester (CFSE) and co-cultured with anti-CD3/CD28-coated beads for 6 days. For analysis, primed T cells were sorted from co-culture as CFSElow and CD123- cells.	peripheral blood CD4 naive T-cell	3
7	GSE109843	T cell activation study 12 (pDC-CpGA; 6d)	Naive CD4 T cells activated by co-culture with CpGA-activated plasmacytoid dendritic cells (pDC). pDCs were FACS-sorted from healthy blood as Lineage (CD3, CD14, CD16, CD19, CD20, CD56)- HLA-DR+ CD11c- CD123+ cells and activated with 5 ug/ml CpGA for 24 h. Naive CD4 T cells were isolated from healthy blood as negative for CD8, CD14, CD16, CD19, CD20, CD25, CD36, CD56, CD61, CD66b, CD123, HLA-DR, TCRγ/δ, glycophorin A and CD45RO, labeled with carboxyfluorescein succinimidyl ester (CFSE) and co-cultured with CpGA-activated pDCs (pDC:T cell ratio 1:6) for 6 days. For analysis, primed T cells were sorted from co-culture as CFSElow and CD123- cells.	peripheral blood CD4 naive T-cell	3	T cell activation study 12 (CD3/CD28; 6d)	Naive CD4 T cells activated by co-culture with anti-CD3/CD28-coated beads. Naive CD4 T cells were isolated from healthy blood as negative for CD8, CD14, CD16, CD19, CD20, CD25, CD36, CD56, CD61, CD66b, CD123, HLA-DR, TCRγ/δ, glycophorin A and CD45RO, labeled with carboxyfluorescein succinimidyl ester (CFSE) and co-cultured with anti-CD3/CD28-coated beads for 6 days. For analysis, primed T cells were sorted from co-culture as CFSElow and CD123- cells.	peripheral blood CD4 naive T-cell	3
8	GSE60482	tofacitinib study 1 (0.1uM)	Tofacinitib (0.1uM) treated in vitro activated T-cells obtained from blood of a healthy donor. Naïve CD4+CD45RA+CD45RO− T cell population was FACS-sorted from peripheral blood mononuclear cells isolated by Ficoll paque. T-cells were activated by plate-bound anti-CD3/anti-CD28 in supplemented RPMI 1640 medium containing 10% FCS for 3 days and cultured in the presence of IL-2 for 1 day. During T cell activation, cells were treated with 0.1uM tofacitinib, a Janus kinase inhibitor.	peripheral blood CD4 activated T-cell (unspecified)	4	untreated activated T-cell sample	In vitro activated T-cells obtained from blood of a healthy donor. Naïve CD4+CD45RA+CD45RO− T cell population was FACS-sorted from peripheral blood mononuclear cells isolated by Ficoll paque. T-cells were activated by plate-bound anti-CD3/anti-CD28 in supplemented RPMI 1640 medium containing 10% FCS for 3 days and cultured in the presence of IL-2 for 1 day.	peripheral blood CD4 activated T-cell (unspecified)	4
9	GSE132270	IL-15 study 1 (10d)	Adherent monocytes isolated from peripheral blood of three donors were treated with IL-15 for 10 days.	peripheral blood monocyte	3	IL-15 study 1 (1d)	Adherent monocytes isolated from peripheral blood of three donors were treated with IL-15 for 1 day.	peripheral blood monocyte	3
10	GSE116405	M. tuberculosis study 6 (live MTB; 72h)	Monocyte-derived dendritic cells isolated from healthy donors were stimulated with live Mycobacterium tuberculosis (MTB) for 72 hours. Monocytes were differentiated into DCs by adding rhIL-4 (20 ng/mL) and rhGM-CSF (20 ng/mL) in the cell culture medium and infected with MTB at a multiplicity of infection (MOI) of 1:1.	monocyte derived dendritic cell	3	M. tuberculosis study 6 (live MTB; 2h)	Monocyte-derived dendritic cells isolated from healthy donors were stimulated with live Mycobacterium tuberculosis (MTB) for 2 hours. Monocytes were differentiated into DCs by adding rhIL-4 (20 ng/mL) and rhGM-CSF (20 ng/mL) in the cell culture medium and infected with MTB at a multiplicity of infection (MOI) of 1:1.	monocyte derived dendritic cell	4
11	GSE125974	Epstein-Barr virus study 1 (28d)	Primary human resting B lymphocytes infected with B95.8 Epstein-Bar virus for 28 days.	B-cell	3	Epstein-Barr virus study 1 (2d)	Primary human resting B lymphocytes infected with B95.8 Epstein-Bar virus for 2 days.	B-cell	3

12	GSE114065	T-cell activation study 18 (allergic; activation)	Naive CD4+ T-cells (CD3+CD4+CD25−CCR7+CD127−) isolated from the blood of children with immunoglobulin E (IgE)-mediated food allergy. T cells were activated with anti-CD3/anti-CD28 antibodies for 72 hours.	peripheral blood CD4 naive T-cell	32	T-cell study 20 (allergic)	Naive CD4+ T-cells (CD3+CD4+CD25−CCR7+CD127−) isolated from the blood of children with immunoglobulin E (IgE)-mediated food allergy. Cells were left resting in media alone for 72 hours.	peripheral blood CD4 naive T-cell	31
13	GSE114065	T-cell activation study 18 (non-allergic; activation)	Naive CD4+ T-cells (CD3+CD4+CD25−CCR7+CD127−) isolated from the blood of non-allergic children and activated with anti-CD3/anti-CD28 antibodies for 72 hours.	peripheral blood CD4 naive T-cell	17	T-cell study 20 (non-allergic)	Naive CD4+ T-cells (CD3+CD4+CD25−CCR7+CD127−) isolated from the blood of non-allergic children. Cells were left resting in media alone for 72 hours.	peripheral blood CD4 naive T-cell	18
14	GSE114065	T-cell activation study 18 (resolved; activation)	Naive CD4+ T-cells (CD3+CD4+CD25−CCR7+CD127−) isolated from the blood of children, which had outgrown their food allergy. T cells were activated with anti-CD3/anti-CD28 antibodies for 72 hours.	peripheral blood CD4 naive T-cell	19	T-cell study 20 (resolved)	Naive CD4+ T-cells (CD3+CD4+CD25−CCR7+CD127−) isolated from the blood of children, which had outgrown their food allergy. Cells were left resting in media alone for 72 hours.	peripheral blood CD4 naive T-cell	17
15	GSE116405	M. tuberculosis study 6 (live MTB; 48h)	Monocyte-derived dendritic cells isolated from healthy donors were stimulated with live Mycobacterium tuberculosis (MTB) for 48 hours. Monocytes were differentiated into DCs by adding rhIL-4 (20 ng/mL) and rhGM-CSF (20 ng/mL) in the cell culture medium and infected with MTB at a multiplicity of infection (MOI) of 1:1.	monocyte derived dendritic cell	5	M. tuberculosis study 6 (live MTB; 2h)	Monocyte-derived dendritic cells isolated from healthy donors were stimulated with live Mycobacterium tuberculosis (MTB) for 2 hours. Monocytes were differentiated into DCs by adding rhIL-4 (20 ng/mL) and rhGM-CSF (20 ng/mL) in the cell culture medium and infected with MTB at a multiplicity of infection (MOI) of 1:1.	monocyte derived dendritic cell	4
16	GSE125974	Epstein-Barr virus study 1 (28d)	Primary human resting B lymphocytes infected with B95.8 Epstein-Bar virus for 28 days.	B-cell	3	Epstein-Barr virus study 1 (4d)	Primary human resting B lymphocytes infected with B95.8 Epstein-Bar virus for 4 days.	B-cell	3
17	GSE125974	Epstein-Barr virus study 1 (4d)	Primary human resting B lymphocytes infected with B95.8 Epstein-Bar virus for 4 days.	B-cell	3	Epstein-Barr virus study 1 (0d)	Primary human resting B lymphocytes infected with B95.8 Epstein-Bar virus for 0 day.	B-cell	3
18	GSE132270	IL-15 study 1 (6d)	Adherent monocytes isolated from peripheral blood of three donors were treated with IL-15 for 6 days.	peripheral blood monocyte	3	IL-15 study 1 (1d)	Adherent monocytes isolated from peripheral blood of three donors were treated with IL-15 for 1 day.	peripheral blood monocyte	3
19	GSE125974	Epstein-Barr virus study 1 (14d)	Primary human resting B lymphocytes infected with B95.8 Epstein-Bar virus for 14 days.	B-cell	3	Epstein-Barr virus study 1 (2d)	Primary human resting B lymphocytes infected with B95.8 Epstein-Bar virus for 2 days.	B-cell	3
20	GSE134416	influenza vaccine study 5 (young; quiescent Tfh cell; 7d)	Quiescent CD4 follicular helper T-cells isolated from blood samples of young adults (30-40 years old) 7 days after vaccination with an influenza vaccine. Cells were isolated by Ficoll gradient centrifugation from buffy coat samples and sorted as CD4+CXCR5+PD1+ICOS-CD38-. Participants had not received any influenza vaccine in the prior 6 months. They were excluded if they had contraindications to influenza vaccine, active substance abuse, HIV/AIDS, clinically active malignancy, immunomodulatory medication need (i.e. chemotherapy, corticosteroids), or active illness (i.e. active respiratory tract infections).	peripheral blood CD4 follicular helper T-cell; Synonym: T-follicular helper cell. A CD4-positive alpha-beta T cell located in follicles of tonsils, that is typically described as CXCR5-positive, CCR7-negative, BCL6-high, ICOS-high and PD1-high, and stimulates follicular B cells to undergo class-switching and antibody production.	5	T-cell study 24 (young; quiescent Tfh cell; baseline)	Quiescent CD4 follicular helper T-cells isolated from blood samples of young adults (30-40 years old) before vaccination (baseline). Cells were isolated by Ficoll gradient centrifugation from buffy coat samples and sorted as CD4+CXCR5+PD1+ICOS-CD38-. Participants had not received any influenza vaccine in the prior 6 months. They were excluded if they had contraindications to influenza vaccine, active substance abuse, HIV/AIDS, clinically active malignancy, immunomodulatory medication need (i.e. chemotherapy, corticosteroids), or active illness (i.e. active respiratory tract infections).	peripheral blood CD4 follicular helper T-cell; Synonym: T-follicular helper cell. A CD4-positive alpha-beta T cell located in follicles of tonsils, that is typically described as CXCR5-positive, CCR7-negative, BCL6-high, ICOS-high and PD1-high, and stimulates follicular B cells to undergo class-switching and antibody production.	5
21	GSE125974	Epstein-Barr virus study 1 (21d)	Primary human resting B lymphocytes infected with B95.8 Epstein-Bar virus for 21 days.	B-cell	3	Epstein-Barr virus study 1 (2d)	Primary human resting B lymphocytes infected with B95.8 Epstein-Bar virus for 2 days.	B-cell	3
22	GSE94099	BI-3802 study 1 (500nM; 168h; Farage)	Samples of lymphoma cell line Farage that was treated with degrader of the transcription factor BCL6 (BI-3802, 500 nM) for 168 hours (7 days). Cells were split once after 3 days to 1 million cells per ml, and fresh medium with BI-3802 was added. Cells were cultured in RPMI.	Farage; Human metastatic lymphoma cell line derived from a lymph node of an adult female Caucasian patient with non-Hodgkin´s lymphoma (B-NHL, diffuse large cell type). Established in 1990. Synonyms:FARAGE; Farage OL; Farage Original Line	3	BI-3802 study 1 (500nM; 20h; Farage)	Samples of lymphoma cell line Farage that was treated with degrader of the transcription factor BCL6 (BI-3802, 500 nM) for 20 hours. Cells were cultured in RPMI.	Farage; Human metastatic lymphoma cell line derived from a lymph node of an adult female Caucasian patient with non-Hodgkin´s lymphoma (B-NHL, diffuse large cell type). Established in 1990. Synonyms:FARAGE; Farage OL; Farage Original Line	3
23	GSE116405	M. tuberculosis study 6 (live MTB; 18h)	Monocyte-derived dendritic cells isolated from healthy donors were stimulated with live Mycobacterium tuberculosis (MTB) for 18 hours. Monocytes were differentiated into DCs by adding rhIL-4 (20 ng/mL) and rhGM-CSF (20 ng/mL) in the cell culture medium and infected with MTB at a multiplicity of infection (MOI) of 1:1.	monocyte derived dendritic cell	5	M. tuberculosis study 6 (live MTB; 2h)	Monocyte-derived dendritic cells isolated from healthy donors were stimulated with live Mycobacterium tuberculosis (MTB) for 2 hours. Monocytes were differentiated into DCs by adding rhIL-4 (20 ng/mL) and rhGM-CSF (20 ng/mL) in the cell culture medium and infected with MTB at a multiplicity of infection (MOI) of 1:1.	monocyte derived dendritic cell	4
24	GSE125974	Epstein-Barr virus study 1 (7d)	Primary human resting B lymphocytes infected with B95.8 Epstein-Bar virus for 7 days.	B-cell	3	Epstein-Barr virus study 1 (2d)	Primary human resting B lymphocytes infected with B95.8 Epstein-Bar virus for 2 days.	B-cell	3
25	GSE116405	M. tuberculosis study 6 (live MTB; 18h)	Monocyte-derived dendritic cells isolated from healthy donors were stimulated with live Mycobacterium tuberculosis (MTB) for 18 hours. Monocytes were differentiated into DCs by adding rhIL-4 (20 ng/mL) and rhGM-CSF (20 ng/mL) in the cell culture medium and infected with MTB at a multiplicity of infection (MOI) of 1:1.	monocyte derived dendritic cell	5	uninfected dendritic cell sample (18 h)	Monocyte-derived dendritic cells isolated from healthy donors and cultivated in vitro for 18 hours. Monocytes were differentiated into DCs by adding rhIL-4 (20 ng/mL) and rhGM-CSF (20 ng/mL) in the cell culture medium.	monocyte derived dendritic cell	5

26	GSE144353	T-cell study 26 (activated; metformin; 2-deoxyglucose)	Peripheral blood CD4 T-cells that were activated with anti-CD3/CD28 and treated with 2-deoxyglucose (1 mM) and metformin (5 mM) for 24 hours. Cells were isolated from whole blood with 90% post isolation purity. Cells were cultured at 37°C and at 5% CO2 in complete RPMI 1640 supplemented with 10% FBS, 10 mM HEPES, 1 mM sodium pyruvate, 13 nonessential amino acids, and 500 mM GlutaMAX.	peripheral blood CD4 activated T-cell (unspecified)	5	T-cell study 26 (activated; mock)	Mock (water) treated peripheral blood CD4 T-cells that were activated with anti-CD3/CD28 for 24 hours. Cells were isolated from whole blood with 90% post isolation purity. Cells were cultured at 37°C and at 5% CO2 in complete RPMI 1640 supplemented with 10% FBS, 10 mM HEPES, 1 mM sodium pyruvate, 13 nonessential amino acids, and 500 mM GlutaMAX.	peripheral blood CD4 activated T-cell (unspecified)	9
27	GSE116405	M. tuberculosis study 6 (live MTB; 48h)	Monocyte-derived dendritic cells isolated from healthy donors were stimulated with live Mycobacterium tuberculosis (MTB) for 48 hours. Monocytes were differentiated into DCs by adding rhIL-4 (20 ng/mL) and rhGM-CSF (20 ng/mL) in the cell culture medium and infected with MTB at a multiplicity of infection (MOI) of 1:1.	monocyte derived dendritic cell	5	uninfected dendritic cell sample (48 h)	Monocyte-derived dendritic cells isolated from healthy donors and cultivated in vitro for 48 hours. Monocytes were differentiated into DCs by adding rhIL-4 (20 ng/mL) and rhGM-CSF (20 ng/mL) in the cell culture medium.	monocyte derived dendritic cell	5
28	GSE114765	T-cell activation study 15 (NAC)	CD8 T cells activated in the presence of N-acetylcysteine (NAC). Naive CD8 T cells were isolated from peripheral blood mononuclear cells by magnetic negative selection as cells negative for CD4, CD16, CD19, CD20, CD36, CD45RO, CD56, CD57, CD235ab, CD244 and TCR gamma/delta. Cells were activated with T-ACT CD3/CD28 Dynabeads (bead/cell ratio 1:2) in the presence of interleukin-2 (IL-2; 10 ng/ml), interleukin-12 (IL-12; 10 ng/ml) and NAC (20 mM) for 8 days. Medium was replaced every 3 days. NAC treatment induced stem cell memory T cell phenotype.	peripheral blood CD8 activated T-cell (unspecified)	6	T-cell activation study 15 (untreated)	CD8 T cells activated without any additional treatment. Naive CD8 T cells were isolated from peripheral blood mononuclear cells by magnetic negative selection as cells negative for CD4, CD16, CD19, CD20, CD36, CD45RO, CD56, CD57, CD235ab, CD244 and TCR gamma/delta. Cells were activated with T-ACT CD3/CD28 Dynabeads (bead/cell ratio 1:2) in the presence of interleukin-2 (IL-2; 10 ng/ml) and interleukin-12 (IL-12; 10 ng/ml) for 8 days. Medium was replaced every 3 days.	peripheral blood CD8 activated T-cell (unspecified)	6
29	GSE77808	NK cell study 1 (IL-15 withdrawn; polysomal)	Peripheral blood natural killer (NK) cells activated with recombinant human IL-15 (18.3 ng/mL) in IMDM medium with 10% human AB serum for 48 hours collected 24 hours after IL-15 withdrawal. Polysome-associated RNA (>2 ribosomes) was isolated from total cytosolic RNA using a sucrose gradient. Primary NK cells had been sorted to >98% purity from T-cells-depleted buffy coats by negative magnetic selection.	peripheral blood natural killer cell	4	NK cell study 1 (IL-2 withdrawn; polysomal)	Peripheral blood natural killer (NK) cells activated with recombinant human IL-2 (18.3 ng/mL) in IMDM medium with 10% human AB serum for 48 hours collected 24 hours after IL-2 withdrawal. Polysome-associated RNA (>2 ribosomes) was isolated from total cytosolic RNA using a sucrose gradient. Primary NK cells had been sorted to >98% purity from T-cells-depleted buffy coats by negative magnetic selection.	peripheral blood natural killer cell	5
30	GSE23391	ovarian tumor study 16	Human epithelial tumor cell samples from the ovary of patients with papillary serous carcinoma. Samples were derived by laser capture microdissection (LCM).	ovary, papillary serous cystadenocarcinoma, NOS	3	normal ovarian surface epithelial cell sample	Human epithelial cell samples from histopathological normal and non-cancerous ovary tissue.	ovarian (surface) epithelium cell	5
31	GSE51952	adefovir study 1 (50uM)	HepG2 cells treated with compound: adefovir (50uM; CAS no.:106941-25-7) for 24 hours. Adefovir is non-hepatotoxic. HepG2 cells were treated with the IC20 concentration measured after 24 hours.	Hep-G2; Human primary cancer cell line derived from the liver of a patient with hepatocellular carcinoma.	3	vehicle (DMSO) treated HepG2 sample	HepG2 cells treated with DMSO (0.5% v/v) as solvent control for 24 hours.	Hep-G2; Human primary cancer cell line derived from the liver of a patient with hepatocellular carcinoma.	21
32	GSE25417	hepatocyte-like cell differentiation study 1 (10d)	Hepatocyte-like cell differentiated from human pluripotent, embryonic stem cell line (ES, WA09). ES cells were grown in human ES cell media DMEM/F12 and differentiated to hepatic specification stage for 10 days.	hepatic cell (ESC); Hepatic-like cells differentiated from human embryonic stem cells (ESC). This stage is approximately corresponding to hepatic specification. Further differentiation results in immature hepatocyte generation.	3	hepatocyte-like cell differentiation study 1 (5d)	Hepatocyte-like cell differentiated from human pluripotent, embryonic stem cell line (ES, WA09). ES cells were grown in human ES cell media DMEM/F12 and differentiated to definitive endoderm for 5 days.	endoderm - definitive (ESC); Definitive endoderm-like cells differentiated from human embryonic stem cells (ESC). This stage is approximately corresponding to definitive endoderm before which is characterized by a high expression of key hepatic transcription factors, including FOXA2, FOXA3, GATA4, GATA6 and HHEX.	3
33	GSE120721	atopic dermatitis study 21 (lesional; whole skin)	Lesional full thickness skin samples isolated from patient with moderate-to-severe atopic dermatitis by laser capture microdissection. Patients' cohort characteristics: 3 males and 2 females; age 27-59 years (mean age: 39.4 years); SCORing of Atopic Dermatitis index (SCORAD) ranging from 45-65; total IgE: 14-1821 kU/l; eosinophilic count: 1.4-11.8 %.	skin	5	normal skin tissue	Full thickness skin samples isolated from healthy subjects by laser capture microdissection.	skin	6
34	GSE12108	F. tularensis study 1 (novicida)	Peripheral blood monocytes infected with the Francisella tularensis subspecies novicida isolate U112 (100 MOI) for 24 hours.	monocyte	4	uninfected peripheral blood monocyte sample	uninfected peripheral blood monocyte sample	monocyte	6
35	GSE59307	cutaneous T-cell lymphoma study 1 (plaque phase)	Lesional skin biopsies from patients with cutaneous T-cell lymphoma in the plaque phase.	cutaneous T-cell lymphoma, NOS	7	normal skin tissue	Skin biopsies from healthy individuals.	skin	8
36	GSE107361	atopic dermatitis study 12 (lesional; adults)	Lesional skin biopsy samples from adult patients (age range 18-73 years) with long-standing atopic dermatitis.	skin	20	normal skin tissue (adults)	Skin biopsy samples from healthy adults (age range 38–57 years).	skin	11

37	GSE41296	formaldehyde study 9 (200µM)	TK6 cells treated with 200µM formaldehyde for 24h.	LCL (hereditary spherocytosis); B-lymphoblastoid cell line derived from the spleen of a male patient with hereditary spherocytosis.	3	untreated TK6 cell sample	Untreated TK6 cell line. Cells where left untreated for 24h before harvesting.	LCL (hereditary spherocytosis); B-lymphoblastoid cell line derived from the spleen of a male patient with hereditary spherocytosis.	15
38	GSE40889	asthma study 33 (nonallergic asthma; PBMC; antiCD3 and antiCD28)	Peripheral blood mononuclear cell (PBMC) culture samples after stimulation with anti-CD3 and anti-CD28 for 48 hours. PBMCs were obtained from pediatric patients with nonallergic asthma (NA). PBMCs were isolated from peripheral blood by density-gradient centrifugation and after dilution to 5x10e6 cells/ml PBMCs were cultured in X-Vivo (Lonza) stimulated with plate-bound anti-CD3 (3 ug/ml) and soluble anti-CD28 (1 ug/ml) at 37°C. Inclusion criteria of the NA subjects: asthma symptoms (at least 3 episodes of wheezing during the last year triggered by exercise, infections, stress), diagnosis of recurrent episodes of obstructive bronchitis, naive to steroid medication/without steroids (also inhaled) medication for at least 14 days, without specific IgE and clinical symptoms of allergy. Exclusion criteria: other chronic pulmonary and autoimmune diseases, immunodeficiency, steroids medication, using of antibiotics or probiotics, current infection or infection within 14 days before blood collection.	peripheral blood mononuclear cell	8	asthma study 33 (nonallergic asthma; PBMC)	Unstimulated peripheral blood mononuclear cell (PBMC) culture samples. PBMCs were obtained from pediatric patients with nonallergic asthma (NA). PBMCs were isolated from peripheral blood by density-gradient centrifugation and after dilution to 5x10e6 cells/ml PBMCs were cultured in X-Vivo (Lonza) unstimulated for 48 hours at 37°C. Inclusion criteria of the NA subjects: asthma symptoms (at least 3 episodes of wheezing during the last year triggered by exercise, infections, stress), diagnosis of recurrent episodes of obstructive bronchitis, naive to steroid medication/without steroids (also inhaled) medication for at least 14 days, without specific IgE and clinical symptoms of allergy. Exclusion criteria: other chronic pulmonary and autoimmune diseases, immunodeficiency, steroids medication, using of antibiotics or probiotics, current infection or infection within 14 days before blood collection.		8
39	GSE103906	CAR T cell study 2 (CD8+; BB-z; 72h)	CD8+ T cells expressing BB-z (CD8-alpha, 4-1BB and CD3zeta) CAR (chimeric antigen receptor) collected 72 hours after stimulation with the CD19+ B-cell acute lymphoblastic leukemia cell line NALM-6. CD8+ T cells were purified by gradient centrifugation and negative magnetic selection from blood samples of healthy volunteers. The purified CD8+ T-cells were stimulated with irradiated artificial antigen-presenting cells aAPC/mOKT3 and IL-2 (100 IU/ml) and IL-15 (10 ng/ml). Then the cells were retrovirally transduced with the CAR-expressing plasmids (on days 2, 3 and 4 following the stimulation). The CAR-transduced cells were co-cultured with the CD19+ B-cell acute lymphoblastic leukemia cell line NALM-6 and purified by FACS after 72 hours of co-culture.	peripheral blood CD8 activated T-cell (unspecified)	4	CAR T cell study 2 (CD8+; BB-z; 4h)	CD8+ T cells expressing BB-z (CD8-alpha, 4-1BB and CD3zeta) CAR (chimeric antigen receptor) collected 4 hours after stimulation with the CD19+ B-cell acute lymphoblastic leukemia cell line NALM-6. CD8+ T cells were purified by gradient centrifugation and negative magnetic selection from blood samples of healthy volunteers. The purified CD8+ T-cells were stimulated with irradiated artificial antigen-presenting cells aAPC/mOKT3 and IL-2 (100 IU/ml) and IL-15 (10 ng/ml). Then the cells were retrovirally transduced with the CAR-expressing plasmids (on days 2, 3 and 4 following the stimulation). The CAR-transduced cells were co-cultured with the CD19+ B-cell acute lymphoblastic leukemia cell line NALM-6 and purified by FACS after 4 hours of co-culture.	peripheral blood CD8 activated T-cell (unspecified)	4
40	GSE103906	CAR T cell study 2 (CD8+; 28-z; 24h)	CD8+ T cells expressing 28-z (CD28 + CD3zeta) CAR (chimeric antigen receptor) collected 24 hours after stimulation with the CD19+ B-cell acute lymphoblastic leukemia cell line NALM-6. CD8+ T cells were purified by gradient centrifugation and negative magnetic selection from blood samples of healthy volunteers. The purified CD8+ T-cells were stimulated with irradiated artificial antigen-presenting cells aAPC/mOKT3 and IL-2 (100 IU/ml) and IL-15 (10 ng/ml). Then the cells were retrovirally transduced with the CAR-expressing plasmids (on days 2, 3 and 4 following the stimulation). The CAR-transduced cells were co-cultured with the CD19+ B-cell acute lymphoblastic leukemia cell line NALM-6 and purified by FACS after 24 hours of co-culture.	peripheral blood CD8 activated T-cell (unspecified)	4	CAR T cell study 2 (CD8+; 28-z; 4h)	CD8+ T cells expressing 28-z (CD28 + CD3zeta) CAR (chimeric antigen receptor) collected 4 hours after stimulation with the CD19+ B-cell acute lymphoblastic leukemia cell line NALM-6. CD8+ T cells were purified by gradient centrifugation and negative magnetic selection from blood samples of healthy volunteers. The purified CD8+ T-cells were stimulated with irradiated artificial antigen-presenting cells aAPC/mOKT3 and IL-2 (100 IU/ml) and IL-15 (10 ng/ml). Then the cells were retrovirally transduced with the CAR-expressing plasmids (on days 2, 3 and 4 following the stimulation). The CAR-transduced cells were co-cultured with the CD19+ B-cell acute lymphoblastic leukemia cell line NALM-6 and purified by FACS after 4 hours of co-culture.	peripheral blood CD8 activated T-cell (unspecified)	4
41	GSE103906	CAR T cell study 2 (CD8+; BB-z; 24h)	CD8+ T cells expressing BB-z (CD8-alpha, 4-1BB and CD3zeta) CAR (chimeric antigen receptor) collected 24 hours after stimulation with the CD19+ B-cell acute lymphoblastic leukemia cell line NALM-6. CD8+ T cells were purified by gradient centrifugation and negative magnetic selection from blood samples of healthy volunteers. The purified CD8+ T-cells were stimulated with irradiated artificial antigen-presenting cells aAPC/mOKT3 and IL-2 (100 IU/ml) and IL-15 (10 ng/ml). Then the cells were retrovirally transduced with the CAR-expressing plasmids (on days 2, 3 and 4 following the stimulation). The CAR-transduced cells were co-cultured with the CD19+ B-cell acute lymphoblastic leukemia cell line NALM-6 and purified by FACS after 24 hours of co-culture.	peripheral blood CD8 activated T-cell (unspecified)	4	CAR T cell study 2 (CD8+; BB-z; 4h)	CD8+ T cells expressing BB-z (CD8-alpha, 4-1BB and CD3zeta) CAR (chimeric antigen receptor) collected 4 hours after stimulation with the CD19+ B-cell acute lymphoblastic leukemia cell line NALM-6. CD8+ T cells were purified by gradient centrifugation and negative magnetic selection from blood samples of healthy volunteers. The purified CD8+ T-cells were stimulated with irradiated artificial antigen-presenting cells aAPC/mOKT3 and IL-2 (100 IU/ml) and IL-15 (10 ng/ml). Then the cells were retrovirally transduced with the CAR-expressing plasmids (on days 2, 3 and 4 following the stimulation). The CAR-transduced cells were co-cultured with the CD19+ B-cell acute lymphoblastic leukemia cell line NALM-6 and purified by FACS after 4 hours of co-culture.	peripheral blood CD8 activated T-cell (unspecified)	4
42	E-MTAB-184	ulcerative colitis study 22	Macroscopically maximally inflamed sigmoid colon biopsies derived from patients diagnosed with ulcerative colitis.	sigmoid colon	13	normal hepatic flexure tissue	Hepatic flexure biopsies derived from control healthy patients.	hepatic flexure	20
43	E-MTAB-184	ulcerative colitis study 22	Macroscopically maximally inflamed sigmoid colon biopsies derived from patients diagnosed with ulcerative colitis.	sigmoid colon	13	ulcerative colitis study 18	Macroscopically uninflamed hepatic flexure biopsies derived from patients diagnosed with ulcerative colitis.	hepatic flexure	36
44	GSE60709	atopic dermatitis study 14 (non-lesional)	Epidermal shaves of non-lesional skin of patients suffering from moderate to severe early-onset persistent atopic dermatitis. The patients received no systemic treatment at the time of sampling.	epidermis	7	normal epidermis tissue	Epidermal shaves of healthy volunteers without a self-reported history of atopic asthma, atopic dermatitis or atopic rhinitis.	epidermis	12

45	GSE97075	HIDS study 1 (visit 3; canakinumab)	Whole blood samples collected from HIDS (Hyperimmunoglobulinemia D with periodic fever syndrome) patients during canakinumab treatment (visit 3, between days 2 and 4 of the study). The patients received canakinumab (Ilaris) subcutaneously at a dose of 300 mg (or 4 mg/kg for those patients weighing ≤40 kg) at day 0 and then every 6 weeks. Patients who experienced a flare before week 4 received an additional dose of 150 mg (or 2 mg/kg for patients weighing ≤40 kg) at the time of flare, and thereafter received 450 mg every 6 weeks (or 6 mg/kg every 6 weeks for patients weighing ≤40 kg) starting at week 6. Patients who experienced a flare between weeks 5 and 6 received a rescue medication (NSAIDs or corticosteroids, 0.5 mg/kg for 3 days) and waited up to week 6 to receive canakinumab (450 mg, or 6 mg/kg for patients weighing ≤40 kg). Clinical trial NCT01303380.	blood	9	HIDS study 1 (baseline)	Whole blood samples collected from HIDS (Hyperimmunoglobulinemia D with periodic fever syndrome) patients during active disease at baseline (visit 2, after anakinra withdrawal, day 0 of the study). Clinical trial NCT01303380.	blood	9
46	GSE97075	HIDS study 1 (visit 4; canakinumab)	Whole blood samples collected from HIDS (Hyperimmunoglobulinemia D with periodic fever syndrome) patients during canakinumab treatment (visit 4, between days 13 and 16 of the study). The patients received canakinumab (Ilaris) subcutaneously at a dose of 300 mg (or 4 mg/kg for those patients weighing ≤40 kg) at day 0 and then every 6 weeks. Patients who experienced a flare before week 4 received an additional dose of 150 mg (or 2 mg/kg for patients weighing ≤40 kg) at the time of flare, and thereafter received 450 mg every 6 weeks (or 6 mg/kg every 6 weeks for patients weighing ≤40 kg) starting at week 6. Patients who experienced a flare between weeks 5 and 6 received a rescue medication (NSAIDs or corticosteroids, 0.5 mg/kg for 3 days) and waited up to week 6 to receive canakinumab (450 mg, or 6 mg/kg for patients weighing ≤40 kg). Clinical trial NCT01303380.	blood	8	HIDS study 1 (baseline)	Whole blood samples collected from HIDS (Hyperimmunoglobulinemia D with periodic fever syndrome) patients during active disease at baseline (visit 2, after anakinra withdrawal, day 0 of the study). Clinical trial NCT01303380.	blood	9
47	GSE97075	HIDS study 1 (visit 3; canakinumab)	Whole blood samples collected from HIDS (Hyperimmunoglobulinemia D with periodic fever syndrome) patients during canakinumab treatment (visit 3, between days 2 and 4 of the study). The patients received canakinumab (Ilaris) subcutaneously at a dose of 300 mg (or 4 mg/kg for those patients weighing ≤40 kg) at day 0 and then every 6 weeks. Patients who experienced a flare before week 4 received an additional dose of 150 mg (or 2 mg/kg for patients weighing ≤40 kg) at the time of flare, and thereafter received 450 mg every 6 weeks (or 6 mg/kg every 6 weeks for patients weighing ≤40 kg) starting at week 6. Patients who experienced a flare between weeks 5 and 6 received a rescue medication (NSAIDs or corticosteroids, 0.5 mg/kg for 3 days) and waited up to week 6 to receive canakinumab (450 mg, or 6 mg/kg for patients weighing ≤40 kg). Clinical trial NCT01303380.	blood	9	normal blood sample	Whole blood samples collected from healthy volunteers.	blood	15
48	GSE97075	HIDS study 1 (visit 4; canakinumab)	Whole blood samples collected from HIDS (Hyperimmunoglobulinemia D with periodic fever syndrome) patients during canakinumab treatment (visit 4, between days 13 and 16 of the study). The patients received canakinumab (Ilaris) subcutaneously at a dose of 300 mg (or 4 mg/kg for those patients weighing ≤40 kg) at day 0 and then every 6 weeks. Patients who experienced a flare before week 4 received an additional dose of 150 mg (or 2 mg/kg for patients weighing ≤40 kg) at the time of flare, and thereafter received 450 mg every 6 weeks (or 6 mg/kg every 6 weeks for patients weighing ≤40 kg) starting at week 6. Patients who experienced a flare between weeks 5 and 6 received a rescue medication (NSAIDs or corticosteroids, 0.5 mg/kg for 3 days) and waited up to week 6 to receive canakinumab (450 mg, or 6 mg/kg for patients weighing ≤40 kg). Clinical trial NCT01303380.	blood	8	normal blood sample	Whole blood samples collected from healthy volunteers.	blood	15

49	GSE97075	HIDS study 1 (visit 3; canakinumab)	Whole blood samples collected from HIDS (Hyperimmunoglobulinemia D with periodic fever syndrome) patients during canakinumab treatment (visit 3, between days 2 and 4 of the study). The patients received canakinumab (Ilaris) subcutaneously at a dose of 300 mg (or 4 mg/kg for those patients weighing ≤40 kg) at day 0 and then every 6 weeks. Patients who experienced a flare before week 4 received an additional dose of 150 mg (or 2 mg/kg for patients weighing ≤40 kg) at the time of flare, and thereafter received 450 mg every 6 weeks (or 6 mg/kg every 6 weeks for patients weighing ≤40 kg) starting at week 6. Patients who experienced a flare between weeks 5 and 6 received a rescue medication (NSAIDs or corticosteroids, 0.5 mg/kg for 3 days) and waited up to week 6 to receive canakinumab (450 mg, or 6 mg/kg for patients weighing ≤40 kg). Clinical trial NCT01303380.	blood	9	HIDS study 1 (anakinra)	Whole blood samples collected from HIDS (Hyperimmunoglobulinemia D with periodic fever syndrome) patients during anakinra treatment at visit 1 (between days -31 and -2 of the study). Clinical trial NCT01303380.	blood	7

Supplementary Table S2.

Experimental conditions leading to *GPR55* downregulation.

		Experimental group				Control group			
ID	Repository	Stimulus	Description	Anatomical Part or Cell line or Neoplasm	n	Stimulus	Description	Anatomical Part or Cell line or Neoplasm	n
1	GSE65621	IL-6 study 6 (healthy; 50 ng/ml)	CD4 Th0 cells activated and treated with interleukin 6 for 3 days in vitro. Peripheral blood mononuclear cells were prepared from venous blood of healthy female donors by Ficoll-gradient centrifugation. Naïve CD4+CD45RAhighCD45ROlow population was purified by the flow cytometry using FACS and were activated by plate-bound anti-CD3 (5 μg/ml) and anti-CD28 (5 μg/ml) in RPMI medium with 10% (vol/vol) FCS, 2 mM glutamine, 100 IU/ml of penicillin, 0.1 mg/ml of streptomycin and 20mM HEPES buffer, pH 7.2-7.5 and 2 mM β–mercaptoethanol for 3 days. Interleukin 6 was added at concentration 50 ng/ml.	peripheral blood CD4 activated T-cell (unspecified)	4	untreated activated CD4 T-cell sample (heatlhy)	Normal CD4 Th0 cells activated for 3 days in vitro. Peripheral blood mononuclear cells were prepared from venous blood of healthy female donors by Ficoll-gradient centrifugation. Naïve CD4+CD45RAhighCD45ROlow population was purified by the flow cytometry using FACS and were activated by plate-bound anti-CD3 (5 μg/ml) and anti-CD28 (5 μg/ml) in RPMI medium with 10% (vol/vol) FCS, 2 mM glutamine, 100 IU/ml of penicillin, 0.1 mg/ml of streptomycin and 20mM HEPES buffer, pH 7.2-7.5 and 2 mM β–mercaptoethanol for 3 days.	peripheral blood CD4 activated T-cell (unspecified)	4
2	GSE103147	pulmonary tuberculosis study 7 (T-cell; 0d; Mtb)	Mycobacterium tuberculosis (Mtb)-stimulated T-cells, from the blood of adolescents with active tuberculosis, at enrollment (0d). Adolescents were included in the South African Adolescent Cohort Study (ACS) only if they had Mtb infection at enrollment, or if they developed an active disease more than 6 months after Mtb infection was first detected. Infection was diagnosed by a positive QuantiFERON TB GOLD In-Tube Assay (QFT; >0.35 IU/l) and/or a positive tuberculin skin test (TST; >10 mm). QFT and/or TST positive adolescents were not given therapy to prevent tuberculosis disease. Active disease was defined as intrathoracic disease, with either two sputum smears positive for acid-fast bacilli, or one positive sputum smear culture confirmed as Mtb complex. Participants were excluded if they developed tuberculosis within 6 months of enrollment or QFT and/or TST conversion, or if they were HIV infected. T-cells were stimulated with live H37Rv Mtb (1x10exp6 CFU/ml) for 12 hours at 37ºC.	peripheral blood T-cell (unspecified)	8	pulmonary tuberculosis study 7 (T-cell; 0d; ESAT-6/CFP-10)	ESAT-6/CFP-10-stimulated T-cells, from the blood of adolescents with active tuberculosis, at enrollment (0d). Adolescents were included in the South African Adolescent Cohort Study (ACS) only if they had Mycobacterium tuberculosis (Mtb) infection at enrollment, or if they developed an active disease more than 6 months after Mtb infection was first detected. Infection was diagnosed by a positive QuantiFERON TB GOLD In-Tube Assay (QFT; >0.35 IU/l) and/or a positive tuberculin skin test (TST; >10 mm). QFT and/or TST positive adolescents were not given therapy to prevent tuberculosis disease. Active disease was defined as intrathoracic disease, with either two sputum smears positive for acid-fast bacilli, or one positive sputum smear culture confirmed as Mtb complex. Participants were excluded if they developed tuberculosis within 6 months of enrollment or QFT and/or TST conversion, or if they were HIV infected. T-cells were stimulated with a peptide pool of ESAT-6 and CFP-10 (1 ug/ml/peptide) for 12 hours at 37ºC, and anti-CD28/anti-CD49d co-stimulatory antibodies (1 ug/ml) were added to the peptide pool.	peripheral blood T-cell (unspecified)	32
3	GSE94859	T-cell activation study 13 (CD4+; CD3/CD28; 16h; PTPN22 1858CC)	Activated CD4+ T-cells isolated from the blood of healthy donors, carriers of PTPN22 non-risk allele 1858CC. The cells were sorted with an influx sorter (BD), using the following markers: CD4+, CD45RA+, CCR7+. After sorting, the cells were activated with anti-CD3/CD28 beads for 16 h.	peripheral blood CD4 naive T-cell	8	T-cell study 11 (CD4+; PTPN22 1858CC)	Naive CD4+ T-cells isolated from the blood of healthy donors, carriers of PTPN22 non-risk allele 1858CC. The cells were sorted with an influx sorter (BD), using the following markers: CD4+, CD45RA+, CCR7+. After sorting, the cells were cultured in medium for 16 h.	peripheral blood CD4 naive T-cell	8
4	GSE103147	pulmonary tuberculosis study 7 (T-cell; 360d; Mtb)	Mycobacterium tuberculosis (Mtb)-stimulated T-cells, from the blood of adolescents with active tuberculosis, at 360 days after enrollment. Adolescents were included in the South African Adolescent Cohort Study (ACS) only if they had Mtb infection at enrollment, or if they developed an active disease more than 6 months after Mtb infection was first detected. Infection was diagnosed by a positive QuantiFERON TB GOLD In-Tube Assay (QFT; >0.35 IU/l) and/or a positive tuberculin skin test (TST; >10 mm). QFT and/or TST positive adolescents were not given therapy to prevent tuberculosis disease. Active disease was defined as intrathoracic disease, with either two sputum smears positive for acid-fast bacilli, or one positive sputum smear culture confirmed as Mtb complex. Participants were excluded if they developed tuberculosis within 6 months of enrollment or QFT and/or TST conversion, or if they were HIV infected. T-cells were stimulated with live H37Rv Mtb (1x10exp6 CFU/ml) for 12 hours at 37ºC.	peripheral blood T-cell (unspecified)	21	pulmonary tuberculosis study 7 (T-cell; 360d)	Ustimulated T-cells, from the blood of adolescents with active tuberculosis, at 360 days after enrollment. Adolescents were included in the South African Adolescent Cohort Study (ACS) only if they had Mycobacterium tuberculosis (Mtb) infection at enrollment, or if they developed an active disease more than 6 months after Mtb infection was first detected. Infection was diagnosed by a positive QuantiFERON TB GOLD In-Tube Assay (QFT; >0.35 IU/l) and/or a positive tuberculin skin test (TST; >10 mm). QFT and/or TST positive adolescents were not given therapy to prevent tuberculosis disease. Active disease was defined as intrathoracic disease, with either two sputum smears positive for acid-fast bacilli, or one positive sputum smear culture confirmed as Mtb complex. Participants were excluded if they developed tuberculosis within 6 months of enrollment or QFT and/or TST conversion, or if they were HIV infected.	peripheral blood T-cell (unspecified)	22

5	GSE103147	M. tuberculosis study 4 (T-cell; 0d; Mtb)	Mycobacterium tuberculosis (Mtb)-stimulated T-cells, from the blood of adolescents with latent Mtb infection, at enrollment (0d). Adolescents were included in the South African Adolescent Cohort Study (ACS) only if they had Mtb infection at enrollment, or if they developed an active disease more than 6 months after Mtb infection was first detected. Infection was diagnosed by a positive QuantiFERON TB GOLD In-Tube Assay (QFT; >0.35 IU/l) and/or a positive tuberculin skin test (TST; >10 mm). QFT and/or TST positive adolescents were not given therapy to prevent tuberculosis disease. Participants were excluded if they developed tuberculosis within 6 months of enrollment or QFT and/or TST conversion, or if they were HIV infected. T-cells were stimulated with live H37Rv Mtb (1x10exp6 CFU/ml) for 12 hours at 37ºC.	peripheral blood T-cell (unspecified)	14	M. tuberculosis study 4 (T-cell; 0d; Ag85)	Ag85A/Ag85B-stimulated T-cells, from the blood of adolescents with latent Mycobacterium tuberculosis (Mtb) infection, at enrollment (0d). Adolescents were included in the South African Adolescent Cohort Study (ACS) only if they had Mtb infection at enrollment, or if they developed an active disease more than 6 months after Mtb infection was first detected. Infection was diagnosed by a positive QuantiFERON TB GOLD In-Tube Assay (QFT; >0.35 IU/l) and/or a positive tuberculin skin test (TST; >10 mm). QFT and/or TST positive adolescents were not given therapy to prevent tuberculosis disease. Participants were excluded if they developed tuberculosis within 6 months of enrollment or QFT and/or TST conversion, or if they were HIV infected. T-cells were stimulated with a peptide pool of Ag85A and Ag85B (1 ug/ml/peptide) for 12 hours at 37ºC, and anti-CD28/anti-CD49d co-stimulatory antibodies (1 ug/ml) were added to the peptide pool.	peripheral blood T-cell (unspecified)	60
6	GSE79272	dendritic cell study 5 (CpG)	Plasmacytoid dendritic cells (pDC) sorted from healthy donor peripheral blood mononuclear cells (PBMCs), that were cultured for 18 hours with 0.5uM CpG C (TLR9 agonist). pDCs were sorted as PI–, Lin1–, HLADR+, CD11c–, BDCA2+, and CD123+.	plasmacytoid dendritic cell	6	unstimulated plasmacytoid dendritic cell sample	Plasmacytoid dendritic cells (pDC) sorted from peripheral blood mononuclear cells (PBMCs). Cells were isolated from healthy adult donors. pDCs were sorted as PI–, Lin1–, HLADR+, CD11c–, BDCA2+, and CD123+.	plasmacytoid dendritic cell	6
7	GSE103147	pulmonary tuberculosis study 7 (T-cell; 360d; Mtb)	Mycobacterium tuberculosis (Mtb)-stimulated T-cells, from the blood of adolescents with active tuberculosis, at 360 days after enrollment. Adolescents were included in the South African Adolescent Cohort Study (ACS) only if they had Mtb infection at enrollment, or if they developed an active disease more than 6 months after Mtb infection was first detected. Infection was diagnosed by a positive QuantiFERON TB GOLD In-Tube Assay (QFT; >0.35 IU/l) and/or a positive tuberculin skin test (TST; >10 mm). QFT and/or TST positive adolescents were not given therapy to prevent tuberculosis disease. Active disease was defined as intrathoracic disease, with either two sputum smears positive for acid-fast bacilli, or one positive sputum smear culture confirmed as Mtb complex. Participants were excluded if they developed tuberculosis within 6 months of enrollment or QFT and/or TST conversion, or if they were HIV infected. T-cells were stimulated with live H37Rv Mtb (1x10exp6 CFU/ml) for 12 hours at 37ºC.	peripheral blood T-cell (unspecified)	21	pulmonary tuberculosis study 7 (T-cell; 360d; ESAT-6/CFP-10)	ESAT-6/CFP-10-stimulated T-cells, from the blood of adolescents with active tuberculosis, at 360 days after enrollment. Adolescents were included in the South African Adolescent Cohort Study (ACS) only if they had Mycobacterium tuberculosis (Mtb) infection at enrollment, or if they developed an active disease more than 6 months after Mtb infection was first detected. Infection was diagnosed by a positive QuantiFERON TB GOLD In-Tube Assay (QFT; >0.35 IU/l) and/or a positive tuberculin skin test (TST; >10 mm). QFT and/or TST positive adolescents were not given therapy to prevent tuberculosis disease. Active disease was defined as intrathoracic disease, with either two sputum smears positive for acid-fast bacilli, or one positive sputum smear culture confirmed as Mtb complex. Participants were excluded if they developed tuberculosis within 6 months of enrollment or QFT and/or TST conversion, or if they were HIV infected. T-cells were stimulated with a peptide pool of ESAT-6 and CFP-10 (1 ug/ml/peptide) for 12 hours at 37ºC, and anti-CD28/anti-CD49d co-stimulatory antibodies (1 ug/ml) were added to the peptide pool.	peripheral blood T-cell (unspecified)	23
8	GSE53655	blood transcriptome study 2 (with globin)	Pooled whole blood samples from healthy subjects (n=6). PolyA+ mRNA samples without globin depletion. Samples were measured in several technical replicates.	blood	12	blood transcriptome study 2 (globin depletion)	Pooled whole blood samples from healthy subjects (n=6). PolyA+ mRNA samples depleted of globin RNA (GLOBINclear kit). Samples were measured in several technical replicates.	blood	7
9	GSE103147	M. tuberculosis study 4 (T-cell; 0d; Mtb)	Mycobacterium tuberculosis (Mtb)-stimulated T-cells, from the blood of adolescents with latent Mtb infection, at enrollment (0d). Adolescents were included in the South African Adolescent Cohort Study (ACS) only if they had Mtb infection at enrollment, or if they developed an active disease more than 6 months after Mtb infection was first detected. Infection was diagnosed by a positive QuantiFERON TB GOLD In-Tube Assay (QFT; >0.35 IU/l) and/or a positive tuberculin skin test (TST; >10 mm). QFT and/or TST positive adolescents were not given therapy to prevent tuberculosis disease. Participants were excluded if they developed tuberculosis within 6 months of enrollment or QFT and/or TST conversion, or if they were HIV infected. T-cells were stimulated with live H37Rv Mtb (1x10exp6 CFU/ml) for 12 hours at 37ºC.	peripheral blood T-cell (unspecified)	14	M. tuberculosis study 4 (T-cell; 0d; ESAT-6/CFP-10)	ESAT-6/CFP-10-stimulated T-cells, from the blood of adolescents with latent Mycobacterium tuberculosis (Mtb) infection, at enrollment (0d). Adolescents were included in the South African Adolescent Cohort Study (ACS) only if they had Mtb infection at enrollment, or if they developed an active disease more than 6 months after Mtb infection was first detected. Infection was diagnosed by a positive QuantiFERON TB GOLD In-Tube Assay (QFT; >0.35 IU/l) and/or a positive tuberculin skin test (TST; >10 mm). QFT and/or TST positive adolescents were not given therapy to prevent tuberculosis disease. Participants were excluded if they developed tuberculosis within 6 months of enrollment or QFT and/or TST conversion, or if they were HIV infected. T-cells were stimulated with a peptide pool of ESAT-6 and CFP-10 (1 ug/ml/peptide) for 12 hours at 37ºC, and anti-CD28/anti-CD49d co-stimulatory antibodies (1 ug/ml) were added to the peptide pool.	peripheral blood T-cell (unspecified)	55

10	GSE103147	M. tuberculosis study 4 (T-cell; 0d; Mtb)	Mycobacterium tuberculosis (Mtb)-stimulated T-cells, from the blood of adolescents with latent Mtb infection, at enrollment (0d). Adolescents were included in the South African Adolescent Cohort Study (ACS) only if they had Mtb infection at enrollment, or if they developed an active disease more than 6 months after Mtb infection was first detected. Infection was diagnosed by a positive QuantiFERON TB GOLD In-Tube Assay (QFT; >0.35 IU/l) and/or a positive tuberculin skin test (TST; >10 mm). QFT and/or TST positive adolescents were not given therapy to prevent tuberculosis disease. Participants were excluded if they developed tuberculosis within 6 months of enrollment or QFT and/or TST conversion, or if they were HIV infected. T-cells were stimulated with live H37Rv Mtb (1x10exp6 CFU/ml) for 12 hours at 37ºC.	peripheral blood T-cell (unspecified)	14	M. tuberculosis study 4 (T-cell; 0d)	Ustimulated T-cells, from the blood of adolescents with latent Mycobacterium tuberculosis (Mtb) infection, at enrollment (0d). Adolescents were included in the South African Adolescent Cohort Study (ACS) only if they had Mtb infection at enrollment, or if they developed an active disease more than 6 months after Mtb infection was first detected. Infection was diagnosed by a positive QuantiFERON TB GOLD In-Tube Assay (QFT; >0.35 IU/l) and/or a positive tuberculin skin test (TST; >10 mm). QFT and/or TST positive adolescents were not given therapy to prevent tuberculosis disease. Participants were excluded if they developed tuberculosis within 6 months of enrollment or QFT and/or TST conversion, or if they were HIV infected.	peripheral blood T-cell (unspecified)	65
11	GSE103147	pulmonary tuberculosis study 7 (T-cell; 540d; Mtb)	Mycobacterium tuberculosis (Mtb)-stimulated T-cells, from the blood of adolescents with active tuberculosis, at 540 days after enrollment. Adolescents were included in the South African Adolescent Cohort Study (ACS) only if they had Mtb infection at enrollment, or if they developed an active disease more than 6 months after Mtb infection was first detected. Infection was diagnosed by a positive QuantiFERON TB GOLD In-Tube Assay (QFT; >0.35 IU/l) and/or a positive tuberculin skin test (TST; >10 mm). QFT and/or TST positive adolescents were not given therapy to prevent tuberculosis disease. Active disease was defined as intrathoracic disease, with either two sputum smears positive for acid-fast bacilli, or one positive sputum smear culture confirmed as Mtb complex. Participants were excluded if they developed tuberculosis within 6 months of enrollment or QFT and/or TST conversion, or if they were HIV infected. T-cells were stimulated with live H37Rv Mtb (1x10exp6 CFU/ml) for 12 hours at 37ºC.	peripheral blood T-cell (unspecified)	21	pulmonary tuberculosis study 7 (T-cell; 540d; Ag85)	Ag85A/Ag85B-stimulated T-cells, from the blood of adolescents with active tuberculosis, at 540 days after enrollment. Adolescents were included in the South African Adolescent Cohort Study (ACS) only if they had Mycobacterium tuberculosis (Mtb) infection at enrollment, or if they developed an active disease more than 6 months after Mtb infection was first detected. Infection was diagnosed by a positive QuantiFERON TB GOLD In-Tube Assay (QFT; >0.35 IU/l) and/or a positive tuberculin skin test (TST; >10 mm). QFT and/or TST positive adolescents were not given therapy to prevent tuberculosis disease. Active disease was defined as intrathoracic disease, with either two sputum smears positive for acid-fast bacilli, or one positive sputum smear culture confirmed as Mtb complex. Participants were excluded if they developed tuberculosis within 6 months of enrollment or QFT and/or TST conversion, or if they were HIV infected. T-cells were stimulated with a peptide pool of Ag85A and Ag85B (1 ug/ml/peptide) for 12 hours at 37ºC, and anti-CD28/anti-CD49d co-stimulatory antibodies (1 ug/ml) were added to the peptide pool.	peripheral blood T-cell (unspecified)	17
12	GSE86884	kidney transplantation study 33 (post-transplant; 1wk)	Peripheral blood mononuclear cells (PBMCs) from whole blood were isolated from patients (n=31) after kidney allograft transplantation (1 week) and after the start of immunosuppressive drugs. 1 patient had simultaneous pancreas and kidney transplantation. All adult kidney recipients received thymoglobulin induction and maintenance therapy with tacrolimus or cyclosporine, with mycophenolate and short course steroids to days 5–7 post-transplant. None of the patients in the study developed acute rejection (AR) or chronic allograft dysfunction (CGD) within the 6 month study period. Patients characteristics: 9 women and 22 men; race: Caucasian/White (26), African American/Black (2), American Indian/AlaskaNative (3).The RNA integrity number (RIN) values for all samples were >6.	peripheral blood mononuclear cell	31	kidney transplantation study 33 (pre-transplant)	Peripheral blood mononuclear cells (PBMCs) from whole blood were isolated from patients (n=32) before transplantation of kidney due to end stage renal disease. 1 patient was before simultaneous pancreas and kidney transplantation. Four of the patients received tacrolimus or cyclosporine prior to transplantation. Five patients were receiving steroids and 9 were receiving mycophenolate at baseline for underlying disease. The subjects had no rejection or any previous rejection at time of sample collection. Patients characteristics: 9 women and 23 men; age: 48.2 ± 14.4y; race: Caucasian/White (27), African American/Black (2), American Indian/AlaskaNative (3); transplant type: kidney (31) or simultaneous pancreas and kidney (1); primary disease: diabetes (7), glomerular disease (8), hypertension (3), polycystic disease (6), other (8).The RNA integrity number (RIN) values for all samples were >6.	peripheral blood mononuclear cell	32
13	GSE65621	IL-6 study 6 (STAT1 GOF; 50 ng/ml)	CD4 Th0 cells activated and treated with interleukin 6 for 3 days in vitro. Peripheral blood mononuclear cells were prepared from venous blood of female donors with STAT1 gain-of-function (GOF) mutation by Ficoll-gradient centrifugation. Naïve CD4+CD45RAhighCD45ROlow population was purified by the flow cytometry using FACS and were activated by plate-bound anti-CD3 (5 μg/ml) and anti-CD28 (5 μg/ml) in RPMI medium with 10% (vol/vol) FCS, 2 mM glutamine, 100 IU/ml of penicillin, 0.1 mg/ml of streptomycin and 20mM HEPES buffer, pH 7.2-7.5 and 2 mM β–mercaptoethanol for 3 days. Interleukin 6 was added at concentration 50 ng/ml.	peripheral blood CD4 activated T-cell (unspecified)	5	untreated activated CD4 T-cell sample (STAT1 GOF)	STAT1 GOF CD4 Th0 cells activated for 3 days in vitro. Peripheral blood mononuclear cells were prepared from venous blood of female donors with STAT1 gain-of-function (GOF) mutation by Ficoll-gradient centrifugation. Naïve CD4+CD45RAhighCD45ROlow population was purified by the flow cytometry using FACS and were activated by plate-bound anti-CD3 (5 μg/ml) and anti-CD28 (5 μg/ml) in RPMI medium with 10% (vol/vol) FCS, 2 mM glutamine, 100 IU/ml of penicillin, 0.1 mg/ml of streptomycin and 20mM HEPES buffer, pH 7.2-7.5 and 2 mM β–mercaptoethanol for 3 days.	peripheral blood CD4 activated T-cell (unspecified)	5
14	GSE60424	sepsis study 1 (blood)	Whole peripheral blood samples collected from patients with sepsis.	blood	3	normal blood sample	Whole peripheral blood samples collected from healthy subjects.	blood	4
15	GSE118254	systemic lupus erythematosus study 48 (resting naive)	Peripheral blood FACS sorted (CD19+, IgD+, CD27-, MTG-, CD24+, CD38+) resting naive B cells (rN) isolated from African-American female patients with active systemic lupus erythematosus (SLE). (MTG is abbreviation for MitoTracker Green dye).	peripheral blood naive B-cell	9	B-cell study 4 (resting naive)	Peripheral blood FACS sorted (CD19+, IgD+, CD27-, MTG-, CD24+, CD38+) resting naive B cells (rN) isolated from healthy African-American female donors. (MTG is abbreviation for MitoTracker Green dye).	peripheral blood naive B-cell	6
16	GSE86452	T-cell study 25 (Treg; CD161+)	CD161+ regulatory T cells (Treg) isolated from the peripheral blood of healthy adult donors. Cells were sorted by FACS using the following markers: CD4+CD127lowCD25hiCD161+.	peripheral blood CD4 regulatory T-cell (unspecified)	3	T-cell study 25 (Treg; CD161-)	CD161- regulatory T cells (Treg) isolated from the peripheral blood of healthy adult donors. Cells were sorted by FACS using the following markers: CD4+CD127lowCD25hiCD161–.	peripheral blood CD4 regulatory T-cell (unspecified)	4

17	GSE94099	BI-3812 study 1 (500nM; 168h; Farage)	Samples of lymphoma cell line Farage that was treated with inhibitor of the transcription factor BCL6 (BI-3812, 500 nM) for 168 hours (7 days). Cells were split once after 3 days to 1 million cells per ml, and fresh medium with BI-3812 was added. Cells were cultured in RPMI.	Farage	3	BI-3802 study 1 (500nM; 168h; Farage)	Samples of lymphoma cell line Farage that was treated with degrader of the transcription factor BCL6 (BI-3802, 500 nM) for 168 hours (7 days). Cells were split once after 3 days to 1 million cells per ml, and fresh medium with BI-3802 was added. Cells were cultured in RPMI.	Farage	3
18	GSE71645	T-helper activation study 4 (Th2)	T-helper 2 (Th2) polarized cells CD3/CD28-activated and polarized with IL-4 (10 ng/ml) for 72 hours. Naive CD4+ T cells were derived from umbilical cord blood of healthy neonates. Cells were activated with plate-bound anti-CD3 (2.5 µg/ml) and soluble anti-CD28 (500 ng/ml) and polarization was initiated simultaneously with IL-4 and the Th1 neutralizing antibody anti-interferon gamma (1 µg/ml). IL-2 (40U/ml) was added on the second day of culture and polarization was verified by checking the expression of polarization marker genes for Th2.	umbilical cord blood Th2 T-cell	3	T-helper activation study 4 (TH0)	Activated T-cells (Th0) activated with CD3/CD28 for 72 hours. Naive CD4+ T cells were derived from umbilical cord blood of healthy neonates. Cells were activated with plate-bound anti-CD3 (2.5 µg/ml) and soluble anti-CD28 (500 ng/ml) and simultaneously cultured with neutralizing antibodies (anti-interferon γ and anti-IL4) but without polarizing cytokines.	umbilical cord blood CD4 activated T-cell	3
19	PRJEB11844	non-small cell lung cancer study 7 (Treg)	Tumor-infiltrating regulatory CD4+ T-cells isolated from primary tumor tissue of non-small cell lung cancer patients. T-cells were purified from single cell suspensions by Percoll gradient centrifugation and Treg cells were sorted by flow cytometry using the following markers: CD4+/CD25+/CD127-.	tumor derived CD4 regulatory T-cell (unspecified)	8	T-cell study 4 (Treg; blood; healthy)	Regulatory CD4+ T-cells isolated from peripheral blood of healthy donors by fow cytometry using the following markers: CD4+/CD25+/CD127-.	peripheral blood CD4 regulatory T-cell (unspecified)	3
20	GSE53091	MAPK1 depletion study 1	RNAi-mediated gene knockdown of MAPK1 in colorectal cancer cell line SW-480.	SW480	6	mock transfected SW-480 cell sample	SW-480 colorectal cell line sample transduced with non-targeting pLKO_NonTarget vector.	SW480	6
21	GSE53091	RAF1 depletion study 1	RNAi-mediated gene knockdown of RAF1 in colorectal cancer cell line SW-480.	SW480	6	control vector transfected SW-480 cell sample	SW-480 colorectal cell line sample transduced with empty vector pLKO.	SW480	6
22	GSE41825	dendritic cell study 6 (CpG A)	Plasmacytoid dendritic cells (pDCs) collected from blood of healthy donors and treated with CpG-A ODN2216 Class A (TLR9 agonist) for 3 hours. pDCs were sorted as CD303+, CD123+, CD45+, CD69+, CD40+, CD86+.	plasmacytoid dendritic cell	7	dendritic cell study 6 (untreated)	Plasmacytoid dendritic cells (pDCs) collected from blood of healthy donors. pDCs were sorted as CD303+, CD123+, CD45+, CD69+, CD40+, CD86+.	plasmacytoid dendritic cell	8
23	GSE41825	dendritic cell study 6 (gardiquimod; RN486)	Plasmacytoid dendritic cells (pDCs) collected from blood of healthy donors and treated with gardiquimod (TLR7 agonist) and RN486 (Bruton's tyrosine kinase; BTK inhibitor) for 3 hours. pDCs were sorted as CD303+, CD123+, CD45+, CD69+, CD40+, CD86+.	plasmacytoid dendritic cell	4	dendritic cell study 6 (CpG A; RN486)	Plasmacytoid dendritic cells (pDCs) collected from blood of healthy donors and treated with CpG-A ODN2216 Class A (TLR9 agonist) and RN486 (Bruton's tyrosine kinase; BTK inhibitor) for 3 hours. pDCs were sorted as CD303+, CD123+, CD45+, CD69+, CD40+, CD86+.	plasmacytoid dendritic cell	4
24	GSE10767	pulmonary arterial hypertension study 1	EBV-immortalized B cells from BMPR2 mutation (T354G) carriers with clinical pulmonary arterial hypertension.	BMPR2 LCL 264<br>BMPR2 LCL 186<br>BMPR2 LCL 723<br>BMPR2 LCL 266	4	EBV-immortalized B-cell sample	EBV-immortalized B cells from BMPR2 mutation (exon 3 T354G) carriers (all > 60 years old) with no sign of heritable pulmonary arterial hypertension. Asymptomatic status was confirmed in each individual by echocardiography at the time of blood draw.	BMPR2 LCL 172<br>BMPR2 LCL 180<br>BMPR2 LCL 176	3
25	GSE53091	RAF1 depletion study 1	RNAi-mediated gene knockdown of RAF1 in colorectal cancer cell line SW-480.	SW480	6	mock transfected SW-480 cell sample	SW-480 colorectal cell line sample transduced with non-targeting pLKO_NonTarget vector.	SW480	6
26	GSE41825	dendritic cell study 6 (gardiquimod; RN486)	Plasmacytoid dendritic cells (pDCs) collected from blood of healthy donors and treated with gardiquimod (TLR7 agonist) and RN486 (Bruton's tyrosine kinase; BTK inhibitor) for 3 hours. pDCs were sorted as CD303+, CD123+, CD45+, CD69+, CD40+, CD86+.	plasmacytoid dendritic cell	4	dendritic cell study 6 (untreated)	Plasmacytoid dendritic cells (pDCs) collected from blood of healthy donors. pDCs were sorted as CD303+, CD123+, CD45+, CD69+, CD40+, CD86+.	plasmacytoid dendritic cell	8
27	GSE25417	hepatocyte-like cell differentiation study 1 (15d)	Hepatocyte-like cells differentiated from human embryonic stem cells (ES, WA09), 15 days after the onset of differentiation. This stage is approximately corresponding to immature hepatocytes. Human H9 (WA09) ES cells were routinely cultured under low oxygen conditions (4% O2/5% CO2) in human ES cell media (DMEM/F12 medium supplemented with 20% knockout serum replacement, non essential amino acids, glutamine, penicillin/streptomycin and bFGF (4ng/ml) on Matrigel coated plates using MEF-conditioned human ES cell media. Differentiation was initiated by culture for 5 days with 100ng/ml Activin A in RPMI/B27 medium under ambient oxygen/5%CO2 followed by 5 days with 20ng/ml BMP4 /10ng/ml FGF-2 in RPMI/B27 under 4%O2/5%CO2, then 5 days with 20ng/ml HGF in RPMI/B27 supplement under 4%O2/5%CO2.	hepatocyte - immature (WA09)	3	hepatocyte-like cell differentiation study 1 (10d)	Hepatocyte-like cell differentiated from human pluripotent, embryonic stem cell line (ES, WA09). ES cells were grown in human ES cell media DMEM/F12 and differentiated to hepatic specification stage for 10 days.	hepatocyte - immature (WA09)	3
28	GSE25417	hepatocyte-like cell differentiation study 1 (20d; HNF4 depl)	Hepatocyte-like cell differentiated from human pluripotent, embryonic stem cell line (ES, WA09) and transfected with shRNA targeting HNF4A. ES cells were grown in human ES cell media DMEM/F12 and differentiated to mature hepatocytes for 20 days. Moreover hESc was stable transfected with lentivirus expressing an shRNA that efficiently depletes HNF4A.	hepatocyte - immature (WA09)	3	hepatocyte-like cell differentiation study 1 (10d)	Hepatocyte-like cell differentiated from human pluripotent, embryonic stem cell line (ES, WA09). ES cells were grown in human ES cell media DMEM/F12 and differentiated to hepatic specification stage for 10 days.	hepatocyte - immature (WA09)	3

29	GSE102823	CAR T cell study 4 (PSCA-28t28Z; post-infusion)	CD8+ T cells transduced with PSCA-28t28Z (second generation CAR) and isolated 30 days after adoptive transfer into mice bearing HPAC-derived pancreatic tumor. Human peripheral blood mononuclear cells were stimulated with anti-CD3 antibody (OKT3) in presence of IL-2. Two days post-stimulation, T cells were transduced with retroviral vectors encoding PSCA-28t28Z. Cells were cultured for 2 weeks in presence of IL-2 and then transfered into 4-5-week-old male NSG mice. Subcutaneous xenografts were generated by injection of HPAC cells. Once tumors became palpable, mice were treated with CD8+ T cells expressing PSCA-28t28Z. Untransduced CD4+ cells from the same donor were given to each mouse for cytokine support. Spleen-resident human CD8+ T cells were isolated 30 days later using the CD8 MicroBeads (post-infusion samples).	splenic CD8 activated T-cell (unspecified)	3	CAR T cell study 4 (PSCA-28t28Z; pre-infusion)	Primary human CD8+ T cells stimulated ex vivo and transduced to express PSCA-28t28Z (second generation CAR). Human peripheral blood mononuclear cells were stimulated with anti-CD3 antibody (OKT3) in presence of IL-2. Two days post-stimulation, T cells were transduced with retroviral vectors encoding PSCA-28t28Z. Cells were cultured for 2 weeks in presence of IL-2, until collection of samples (pre-infusion samples).	peripheral blood CD8 activated T-cell (unspecified)	3
30	GSE102823	CAR T cell study 4 (GFP; post-infusion)	CD8+ T cells transduced with GFP and isolated 30 days after adoptive transfer into mice bearing HPAC-derived pancreatic tumor. Human peripheral blood mononuclear cells were stimulated with anti-CD3 antibody (OKT3) in presence of IL-2. Two days post-stimulation, T cells were transduced with retroviral vectors encoding GFP as a control. Cells were cultured for 2 weeks in presence of IL-2 and then transfered into 4-5-week-old male NSG mice. Subcutaneous xenografts were generated by injection of HPAC cells. Once tumors became palpable, mice were treated with CD8+ T cells expressing GFP (control group). Untransduced CD4+ cells from the same donor were given to each mouse for cytokine support. Spleen-resident human CD8+ T cells were isolated 30 days later using the CD8 MicroBeads (post-infusion samples).	splenic CD8 activated T-cell (unspecified)	3	CAR T cell study 4 (GFP; pre-infusion)	Primary human CD8+ T cells stimulated ex vivo and transduced to express GFP. Human peripheral blood mononuclear cells were stimulated with anti-CD3 antibody (OKT3) in presence of IL-2. Two days post-stimulation, T cells were transduced with retroviral vectors encoding GFP as a control. Cells were cultured for 2 weeks in presence of IL-2, until collection of samples (pre-infusion samples).	peripheral blood CD8 activated T-cell (unspecified)	3
31	GSE102823	CAR T cell study 4 (PSCA-8t28BBZ; post-infusion)	CD8+ T cells transduced with PSCA-8t28BBZ (third generation CAR) and isolated 30 days after adoptive transfer into mice bearing HPAC-derived pancreatic tumor. Human peripheral blood mononuclear cells were stimulated with anti-CD3 antibody (OKT3) in presence of IL-2. Two days post-stimulation, T cells were transduced with retroviral vectors PSCA-8t28BBZ. Cells were cultured for 2 weeks in presence of IL-2 and then transfered into 4-5-week-old male NSG mice. Subcutaneous xenografts were generated by injection of HPAC cells. Once tumors became palpable, mice were treated with CD8+ T cells expressing PSCA-8t28BBZ. Untransduced CD4+ cells from the same donor were given to each mouse for cytokine support. Spleen-resident human CD8+ T cells were isolated 30 days later using the CD8 MicroBeads (post-infusion samples).	splenic CD8 activated T-cell (unspecified)	3	CAR T cell study 4 (PSCA-8t28BBZ; pre-infusion)	Primary human CD8+ T cells stimulated ex vivo and transduced to express PSCA-8t28BBZ (third generation CAR). Human peripheral blood mononuclear cells were stimulated with anti-CD3 antibody (OKT3) in presence of IL-2. Two days post-stimulation, T cells were transduced with retroviral vectors encoding PSCA-8t28BBZ. Cells were cultured for 2 weeks in presence of IL-2, until collection of samples (pre-infusion samples).	peripheral blood CD8 activated T-cell (unspecified)	3
32	GSE19491	pulmonary tuberculosis study 5 (active; blood; baseline)	Blood samples collected from adult patients with active pulmonary tuberculosis (pTB) that was confirmed by Mycobacterium tuberculosis culture test of either sputum or bronchoalveolar lavage fluid. Samples were obtained before anti-mycobacterial treatment. Patients who were pregnant, immunosuppressed, HIV-positive or had diabetes or an autoimmune disorder were excluded from the study.	blood	7	normal blood sample	Blood samples obtained from healthy donors that were negative for tuberculin skin test (positivity was termed as ≥6 mm if BCG-unvaccinated, ≥15 mm if BCG-vaccinated) and M. tuberculosis antigen-specific interferon-γ release assay (IGRA; Quantiferon Gold In-Tube Assay).	blood	104

Supplementary Table S3.

Experimental conditions promoting LPI accumulation.

		Experimental group				Control group			
ID	Repository	Stimulus	Description	Anatomical Part or Cell line or Neoplasm	n	Stimulus	Description	Anatomical Part or Cell line or Neoplasm	n
1	GSE116405	M. tuberculosis study 6 (live MTB; 18h)	Monocyte-derived dendritic cells isolated from healthy donors were stimulated with live Mycobacterium tuberculosis (MTB) for 18 hours. Monocytes were differentiated into DCs by adding rhIL-4 (20 ng/mL) and rhGM-CSF (20 ng/mL) in the cell culture medium and infected with MTB at a multiplicity of infection (MOI) of 1:1.	monocyte derived dendritic cell	5	uninfected dendritic cell sample (18 h)	Monocyte-derived dendritic cells isolated from healthy donors and cultivated in vitro for 18 hours. Monocytes were differentiated into DCs by adding rhIL-4 (20 ng/mL) and rhGM-CSF (20 ng/mL) in the cell culture medium.	monocyte derived dendritic cell	5
2	GSE47944	psoriasis study 16 (lesional; untreated)	Lesional skin biopsies obtained from psoriatic patients of European descendent (5 males, 3 females; mean age 52 years, range 30-63 years). Patients were not receiving any systemic treatment at the time of visit were recruited. 6 mm full-thickness skin biopsy was obtained from the margin of a lesion.	skin	8	psoriasis study 16 (non-lesional; untreated)	Non-lesional skin biopsies obtained from psoriatic patients of European descendent (5 males, 3 females; mean age 52 years, range 30-63 years). Patients were not receiving any systemic treatment at the time of visit were recruited. 6 mm full-thickness skin biopsy was obtained from non-lesional skin at least 5 cm apart from any psoriatic lesion at the same anatomical site.	skin	8
3	GSE47944	psoriasis study 16 (lesional; untreated)	Lesional skin biopsies obtained from psoriatic patients of European descendent (5 males, 3 females; mean age 52 years, range 30-63 years). Patients were not receiving any systemic treatment at the time of visit were recruited. 6 mm full-thickness skin biopsy was obtained from the margin of a lesion.	skin	8	normal skin tissue (healthy; untreated)	Skin biopsy from healthy subjects. 5 mm full-thickness skin biopsies were obtained from discarded healthy skin from female donors (mean age 51 years, range 36-74 years) undergoing plastic surgery procedures.	skin	5
4	TCGA	TCGA colorectal cancer study 1 (mucinous adenocarcinoma; rect. jun.)	Primary tumor tissue sample obtained from the rectosigmoid junction of patients with mucinous adenocarcinoma.	rectosigmoid junction, mucinous adenocarcinoma	8	adjacent rectosigmoid junction tissue TCGA	Histologically normal rectosigmoid colon tissue sample from patients with colorectal cancer.	rectosigmoid junction	6
5	TCGA	TCGA lung cancer study 1 (micropapillary adenocarcinoma)	Primary tumor tissue samples obtained from patients with lung micropapillary adenocarcinoma.	bronchus or lung, micropapillary carcinoma	3	adjacent lung tissue TCGA	Histological normal lung tissue samples from patients with lung cancer.	lung	106
6	TCGA	TCGA colorectal cancer study 1 (mucinous adenocarcinoma; colon)	Primary tumor tissue sample obtained from the colon of patients with mucinous adenocarcinoma.	colon, mucinous adenocarcinoma	11	adjacent colon tissue TCGA	Histologically normal colon tissue sample from patients with colorectal cancer.	colon	13
7	TCGA	TCGA colorectal cancer study 1 (adenocarcinoma; colon)	Primary tumor tissue sample obtained from the colon of patients with adenocarcinoma.	colon, adenocarcinoma, NOS	87	adjacent colon tissue TCGA	Histologically normal colon tissue sample from patients with colorectal cancer.	colon	13
8	GSE50760	colorectal cancer study 26 (metastatic)	Liver tissue samples derived from the metastatic site of patients with stage IV colorectal adenocarcinoma.	colon, adenocarcinoma, NOS, metastatic	16	adjacent colon epithelium tissue	Adjacent normal colon tissue biopsies from patients with stage IV colorectal adenocarcinoma.	colorectum	18
9	GSE15543	pancreatic islet study 3 (re-differentiated; NIH)	Pancreatic islet cells were expanded for 10 weeks and re-differentiated for 1 week according to National Institutes of Health (NIH) protocol. Re-differentiation phase: Expanded cells were cultured for 1 week in serum-free CMRL-1066 medium supplemented with insulin (10 g/ml), transferrin (5.5 g/ml), sodium selenite (6.7ng/ml).	pancreatic islet cell	4	normal pancreatic islet sample	Pancreatic islets were obtained from seven donors aged between 37 and 70 years and body mass index between 22 and 27. Functional islets were cultured in CMRL 1066 supplemented with 10% FCS, 1% glutamine, 5.6 mM glucose, 1 mM HEPES, 110 U/ml penicillin, and 110 g/ml streptomycin for 7 days or immediately processed after isolation.	pancreatic islet cell	7
10	GSE35493	medulloblastoma study 2	Primary tumor tissue sample from the infratentorial brain of pediatric patients with large-cell anaplastic medulloblastoma.	brain, medulloblastoma, NOS	4	non-tumor brain tissue	Histologically normal brain tissue at rapid autopsy from patients who died from atypical teratoid/rhabdoid tumor.	frontal lobe, occipital lobe, parietal lobe, temporal lobe, brain (encephalon), cerebellum	9
11	GSE31210	lung adenocarcinoma study 6 (EGFR mut)	Primary tumor sample from patients with stage II adenocarcinoma carrying an EGFR mutation.	bronchus or lung, adenocarcinoma, NOS	24	adjacent lung tissue	Adjacent and histologically normal, non-tumorous lung tissue samples from patients with lung adenocarcinoma.	lung	20
12	GSE39612	Merkel cell carcinoma study 3 (primary)	Primary tumor tissue from the skin of patients with Merkel cell carcinoma.	skin, Merkel cell carcinoma	19	normal skin tissue	Normal skin samples from healthy donors.	skin	64
13	GSE18105	colorectal cancer study 25	Human primary colorectal carcinoma sample.	colon, neoplasm, malignant	94	adjacent colon tissue	Normal colon tissue samples adjacent to tumor from patients with colorectal cancer.	colon	17
14	GSE39612	Merkel cell carcinoma study 3 (metastatic)	Metastatic tumor tissue from different metastatic sites (skin, lymph node, parotid gland) of patients with Merkel cell carcinoma of the skin	skin, Merkel cell carcinoma, metastatic	11	normal skin tissue	Normal skin samples from healthy donors.	skin	64
15	GSE21510	colorectal cancer study 4 (non-recurring)	LCM-tumor tissue samples of patients who received resection after diagnosis of primary colorectal cancer. Patients did not develop metastatic recurrence during follow-up after surgery.	colon, neoplasm, malignant	76	adjacent colon tissue (non-recurring)	Histologically normal colon tissue samples from patients with primary colorectal cancer collected after resection. Tissue was further extracted using laser-capture microdissection (LCM). No metastatic recurrence during follow-up.	colon	18
16	GSE21510	colorectal cancer study 4 (recurring)	LCM-tumor tissue samples of patients who received resection after diagnosis of primary colorectal cancer. Patients developed metastatic recurrence during follow-up after surgery.	colon, neoplasm, malignant	24	adjacent colon tissue (recurring)	Histologically normal colon tissue samples from patients with primary colorectal cancer collected after resection. Tissue was further extracted using laser-capture microdissection (LCM). With metastatic recurrence after resection during follow-up.	colon	3

Supplementary Table S4.

Experimental conditions promoting LPI depletion.

		Experimental group				Control group			
ID	Repository	Stimulus	Description	Anatomical Part or Cell line or Neoplasm	n	Stimulus	Description	Anatomical Part or Cell line or Neoplasm	n
1	GSE83100	PAX8 depletion study 2 (FT194; siRNA)	Immortalized fallopian tube secretory epithelial FT194 cell sample harvested 72 hours after PAX8 (transcription factor paired box 8) siRNA transfection. Cells were maintained in DMEM:F12 supplemented with 2% Ultraser G and 1% penicillin/streptomycin.	FT194 Immortalized fallopian tube secretory epithelial cell line initially isolated from primary human fallopian tube tissue. Cells were immortalized by stable expression of human telomerase reverse transcriptase (hTERT) and using a single vector containing SV40 large T and small T antigen, causing inactivation of TP53. Synonyms:FT-194 Cellosaurus code:CVCL_UH58	4	PAX8 depletion study 2 (Kuramochi; siRNA)	High-grade serous ovarian cancer Kuramochi cell sample harvested 72 hours after PAX8 (transcription factor paired box 8) siRNA transfection. Cells were maintained in DMEM:F12 media supplemented with 10% FBS and 1% penicillin/streptomycin.	Kuramochi Metastatic cancer cell line derived from the ascites of a patient with high grade ovarian serous adenocarcinoma. Synonyms:KURAMOCHI Cellosaurus code:CVCL_1345	4
2	GSE83100	PAX8 depletion study 2 (FT246; siRNA)	Immortalized fallopian tube secretory epithelial FT246 cell sample harvested 72 hours after PAX8 (transcription factor paired box 8) siRNA transfection. Cells were maintained in DMEM:F12 supplemented with 2% Ultraser G and 1% penicillin/streptomycin.	FT246 Immortalized fallopian tube secretory epithelial cell line initially isolated from primary human fallopian tube tissue. Cells were immortalized by stable expression of human telomerase reverse transcriptase (hTERT), p53 shRNA, and CDK4 R24C. Cellosaurus code:CVCL_UH61	3	PAX8 depletion study 2 (Kuramochi; siRNA)	High-grade serous ovarian cancer Kuramochi cell sample harvested 72 hours after PAX8 (transcription factor paired box 8) siRNA transfection. Cells were maintained in DMEM:F12 media supplemented with 10% FBS and 1% penicillin/streptomycin.	Kuramochi Metastatic cancer cell line derived from the ascites of a patient with high grade ovarian serous adenocarcinoma. Synonyms:KURAMOCHI Cellosaurus code:CVCL_1345	4
3	GSE83100	PAX8 depletion study 2 (FT33; siRNA)	Immortalized fallopian tube secretory epithelial FT33 cell sample harvested 72 hours after PAX8 (transcription factor paired box 8) siRNA transfection. Cells were maintained in DMEM:F12 supplemented with 2% Ultraser G and 1% penicillin/streptomycin.	FT33-shp53-R24C Immortalized fallopian tube secretory epithelial cell line initially isolated from primary human fallopian tube tissue. Cells were immortalized by stable expression of human telomerase reverse transcriptase (hTERT) and using 2 separate vectors for stable expression of SV40 large T antigen and small T antigen. Synonyms:FT33 Cellosaurus code:CVCL_RK66	4	PAX8 depletion study 2 (Kuramochi; siRNA)	High-grade serous ovarian cancer Kuramochi cell sample harvested 72 hours after PAX8 (transcription factor paired box 8) siRNA transfection. Cells were maintained in DMEM:F12 media supplemented with 10% FBS and 1% penicillin/streptomycin.	Kuramochi Metastatic cancer cell line derived from the ascites of a patient with high grade ovarian serous adenocarcinoma. Synonyms:KURAMOCHI Cellosaurus code:CVCL_1345	4

4	GSE115565	iPSC-derived sensory neuron study 1	Sensory neuron samples obtained by ATCC-BXS0116 induced pluripotent stem cells' (iPSCs) differentiation. Sendai virus reprogrammed iPSCs were initially gained from bone marrow CD34+ cells of a healthy female donor. More details about the differentiation process could be found in the supplementary data.	sensory neuron (ATCC-BXS0116) Sensory neurons differentiated from ATCC-BXS0116 induced pluripotent stem cells (iPSC) that were derived from bone marrow CD34+ cells initially obtained from a healthy 31-year-old Caucasian female donor. Parental cell line:CVCL_0A07: ATCC-BXS0116	4	ATCC-BXS0116 cell sample (iPSC)	Induced pluripotent stem cell (iPSC) ATCC-BXS0116 samples. Sendai virus reprogrammed iPSC were initially gained from bone marrow CD34+ cells of a healthy female donor.	ATCC-BXS0116 Human induced pluripotent stem cells (iPSCs) derived from bone marrow CD34+ cells that were obtained from a healthy 31-year-old Caucasian female donor. Synonyms:ATCC-BXS0116 Cellosaurus code:CVCL_0A07	3
5	GSE76122	gliogenesis study 1 (CNTF; BMP4)	Glial progenitor cells differentiated from neural progenitor cells (NPC), which were isolated from three human fetal brains collected from second trimester-aborted fetuses. To induce differentiation, neurospheres formed from NPC were disaggregated into single cells using accutase and plated for 1 week in 6-well plates previously coated with poly-L-Ornythine (PLO) for 3 h and laminin overnight. The differentiation media consisted of DMEM/F12 supplemented with N2, Glutamax, CNTF (20 ng/mL) and BMP4 (10 ng/mL).	fetal glia progenitor cell	3	normal neural progenitor cell sample	Neural progenitor cells (NPC) were isolated from five human fetal brains collected from second trimester-aborted fetuses. Fetal brains were mechanically dissociated into single cells and seeded in 75-mm tissue culture flasks in Neurobasal Media supplemented with EGF (20 ng/mL), FGF (10 ng/mL), B27, Glutamax and Heparin (2 lg/mL).	neural progenitor cell	7
6	TCGA	TCGA hepatocellular carcinoma study 1 (hepatocellular carcinoma, clear cell type)	Primary tumor tissue samples from the liver of patients with hepatocellular carcinoma (clear cell type).	liver, hepatocellular carcinoma, clear cell type	4	TCGA hepatocellular carcinoma study 1 (combined hepatocellular carcinoma and cholangiocarcinoma)	Primary tumor tissue samples from the liver of patients with hepatocellular carcinoma (clear cell type).	liver, combined hepatocellular carcinoma and cholangiocarcinoma	7
7	GSE57896	adipocyte conversion study 1 (wac; DMSO; 24h)	White adipocytes (WAC) differentiated from human pluripotent, embryonic stem cell line (hESC SA001) were treated with DMSO for 24 hours. hESCs were differentiated as embryoid bodies and then re-plated and passaged to generate mesenchymal progenitor cells (MPCs). MPCs were transduced (programmed) with lentivirus constitutively expressing the lenti-rtTA M2 domain and virus carrying the inducible cDNA transgenes PPARG2 (lenti-PPARG2). All differentiated cells were cultured in media containing adipogenic factors and doxycycline for 7 days. At day 7, when cells were differentiating and adopted to white phenotype, pluripotent stem cell-derived white adipocytes (PSC-WA) received DMSO. At day 8, cell culture was collected for further analysis.	adipocyte - white (ESC) White adipocytes-like cells (WA) differentiated from human pluripotent, embryonic stem cells.	3	mock treated mesenchymal progenitor cell sample (24h)	Differentiated mesenchymal progenitor cells (MPCs) derived from human pluripotent, embryonic stem cell line (hESC SA001) were treated with DMSO for 24 hours. hESCs were differentiated as embryoid bodies and then re-plated and passaged to generate mesenchymal progenitor cells (MPCs). MPCs were mock-transduced with lentivirus constitutively expressing only the lenti-rtTA M2 domain without any target gene. The cells were cultured for 7 days. At day 7, MPCs received DMSO. At day 8, cell culture was collected for further analysis.	mesenchymal progenitor cell (ESC) Differentiated mesenchymal progenitor-like cells (MPC) derived from human pluripotent, embryonic stem cells.	3
8	TCGA	TCGA hepatocellular carcinoma study 1 (hepatocellular carcinoma, NOS)	Primary tumor tissue samples from the liver of patients with hepatocellular carcinoma (NOS).	liver, hepatocellular carcinoma, NOS	354	TCGA hepatocellular carcinoma study 1 (combined hepatocellular carcinoma and cholangiocarcinoma)	Primary tumor tissue samples from the liver of patients with combined hepatocellular carcinoma and cholangiocarcinoma.	liver, combined hepatocellular carcinoma and cholangiocarcinoma	7
9	GSE73502	S. typhimurium study 2 (24h)	Monocyte-derived macrophage sample infected with Salmonella typhimurium for 24 hours. The samples were from healthy male donors, which were not under medication, and which tested negative for all the assessed pathogens. Macrophages were infected at a multiplicity of infection (MOI) of 10:1. After 24 hours in contact with the bacteria, macrophages were washed and cultured for an hour in the presence of 50 ug/ml gentamycin in order to kill all bacteria in the medium. The cells were then washed a second time, and cultured in complete medium with 3 ug/ml gentamycin for an additional 24 hours.	monocyte derived macrophage	4	S. typhimurium study 2 (2h)	Monocyte-derived macrophage sample infected with Salmonella typhimurium for 2 hours. The samples were from healthy male donors, which were not under medication, and which tested negative for all the assessed pathogens. Macrophages were infected at a multiplicity of infection (MOI) of 10:1. After 2 hours in contact with the bacteria, macrophages were washed and cultured for an hour in the presence of 50 ug/ml gentamycin in order to kill all bacteria in the medium. The cells were then washed a second time, and cultured in complete medium with 3 ug/ml gentamycin for an additional 2 hours.	monocyte derived macrophage	60
10	GSE107361	atopic dermatitis study 12 (non-lesional; adults)	Unaffected skin biopsy samples from adult patients (age range 18-73 years) with long-standing atopic dermatitis.	skin	20	atopic dermatitis study 12 (non-lesional; children)	Unaffected buttock skin biopsy samples from pediatric patients (age range 3 months-5 years) with early-onset atopic dermatitis. All patients had moderate-to-severe disease (SCORAD score: mean, 57.8; range, 33-84) with recent-onset (within the previous 6 months). Systemic immunosuppressants within the past 4 weeks, topical steroids or immunomodulators within 1 week, or moisturizers within 12 hours before evaluation were restricted. Patients with active skin infections were excluded.	skin	19

11	GSE112239	breast cancer study 47 (CP Lot VIII culture 3d)	GM-CSF-secreting breast cancer cell line SV-BR-1-GM (Master Cell Bank 2; CP Lot VIII from 4-day culture) obtained after culturing. The SV-BR-1-GM cell line was derived from SV-BR-1 breast cancer cells following stable transfection with CSF2 (encoding human granulocyte-macrophage colony-stimulating factor (GM-CSF)). SV-BR-1-GM cells were lysed after culturing in RPMI-1640 supplemented with 10% FBS and GlutaMAX.	SV-BR-1-GM<br>Human cancer cell line SV-BR-1 modified by stable transfection with CSF2 (encoding human granulocyte macrophage colony-stimulating factor (GM-CSF)). The original cell line SV-BR-1 was derived from a chest wall lesion of a metastatic breast cancer female patient. Parental cell line:CVCL_4U12: SV-BR-1 Synonyms:SVBR-1-GM Cellosaurus code:CVCL_4U13	6	breast cancer study 47 (CP Lot VIII culture 1d)	GM-CSF-secreting breast cancer cell line SV-BR-1-GM (Master Cell Bank 2; CP Lot VIII from 2-day culture) obtained after culturing. The SV-BR-1-GM cell line was derived from SV-BR-1 breast cancer cells following stable transfection with CSF2 (encoding human granulocyte-macrophage colony-stimulating factor (GM-CSF)). SV-BR-1-GM cells were lysed after culturing in RPMI-1640 supplemented with 10% FBS and GlutaMAX.	SV-BR-1-GM<br>Human cancer cell line SV-BR-1 modified by stable transfection with CSF2 (encoding human granulocyte macrophage colony-stimulating factor (GM-CSF)). The original cell line SV-BR-1 was derived from a chest wall lesion of a metastatic breast cancer female patient. Parental cell line:CVCL_4U12: SV-BR-1 Synonyms:SVBR-1-GM Cellosaurus code:CVCL_4U13	3
12	GSE106589	schizophrenia study 18 (neuron)	Induced pluripotent stem cell (iPSC)-derived neurons of childhood-onset schizophrenia (COS) donors. COS was diagnosed using the DSM criteria, and there was no clinical, neuroimaging, pharmacological, or genetic evidence to suggest that COS was a distinct disorder. The cohort consists of medication-free patients. IPSCs were generated from normal skin fibroblasts by transduction with Sendai viruses, followed by differentiation to forebrain neurons for 6 weeks (more details about the differentiation procedure can be found in the original publication).	forebrain neuron (iPSC)<br>Differentiated forebrain neurons derived from induced pluripotent stem cells (iPSC).	23	schizophrenia study 18 (NPC)	Induced pluripotent stem cell (iPSC)-derived neural progenitor cells (NPC) of childhood-onset schizophrenia (COS) donors. COS was diagnosed using the DSM criteria, and there was no clinical, neuroimaging, pharmacological, or genetic evidence to suggest that COS was a distinct disorder. The cohort consists of medication-free patients. IPSCs were generated from normal skin fibroblasts by transduction with Sendai viruses, followed by differentiation to forebrain NPC (more details about the differentiation procedure can be found in the original publication).	forebrain progenitor cell (iPSC)<br>Differentiated forebrain progenitor-like cells derived from induced pluripotent stem cells (iPSC).	23
13		schizophrenia study 13 (NPC; DISC1 exon 2 mut/mut)	Neural progenitor cells (NPC) differentiated from human induced pluripotent stem cell (iPSC) line isogenic to cell line YZ1. This cell line was derived from fibroblast obtained from human fetus. Isogenic cell line was genetically modified using CRISPR-Cas9 method and have frameshift mutation in exon 2 (biallelic) in disrupted in schizophrenia 1 (DISC1) gene. IPSC were then cultured as aggregates in a suspension for 4 days in iPSC media (without FGF2), followed by 2 days in N2 neural induction media. Day 7 aggregates were plated onto Matrigel-coated 6 well plates and maintained in N2 neural induction media, forming neuroepithelial structures. At day 17, mRNA from neural rosettes were isolated.	forebrain progenitor cell (iPSC)<br>Differentiated forebrain progenitor-like cells derived from induced pluripotent stem cells (iPSC).	6	ut) / schizophrenia study 13 (NPC; DISC1 exon 8 wt/mut)	Neural progenitor cells (NPC) differentiated from human induced pluripotent stem cell (iPSC) line isogenic to cell line YZ1. This cell line was derived from fibroblast obtained from human fetus. Isogenic cell line was genetically modified using TALENs and have frameshift mutation in exon 8 (monoallelic) in disrupted in schizophrenia 1 (DISC1) gene. IPSC were then cultured as aggregates in a suspension for 4 days in iPSC media (without FGF2), followed by 2 days in N2 neural induction media. Day 7 aggregates were plated onto Matrigel-coated 6 well plates and maintained in N2 neural induction media, forming neuroepithelial structures. At day 17, mRNA from neural rosettes were isolated.	forebrain progenitor cell (iPSC)<br>Differentiated forebrain progenitor-like cells derived from induced pluripotent stem cells (iPSC).	3
14	GSE57896	adipocyte conversion study 1 (wac; tofacitinib; 24h)	White adipocytes (WAC) differentiated from human pluripotent, embryonic stem cell line (hESC SA001) were treated with tofacitinib for 24 hours. hESCs were differentiated as embryoid bodies and then re-plated and passaged to generate mesenchymal progenitor cells (MPCs). MPCs were transduced (programmed) with lentivirus constitutively expressing the lenti-rtTA M2 domain and virus carrying the inducible cDNA transgenes PPARG2 (lenti-PPARG2). All differentiated cells were cultured in media containing adipogenic factors and doxycycline for 7 days. At day 7, when pluripotent stem cell-derived white adipocytes (PSC-WA) were differentiating, tofacitinib (2 µM) was applied to PSC-WA. At day 8, cell culture was collected for further analysis. Tofacitinib is commercially available potent inhibitor of signaling through Janus Kinase 3 (JAK3). ATC code:L04AA29	adipocyte - white (ESC)<br>White adipocytes-like cells (WA) differentiated from human pluripotent, embryonic stem cells.	3	mock treated mesenchymal progenitor cell sample (24h)	Differentiated mesenchymal progenitor cells (MPCs) derived from human pluripotent, embryonic stem cell line (hESC SA001) were treated with DMSO for 24 hours. hESCs were differentiated as embryoid bodies and then re-plated and passaged to generate mesenchymal progenitor cells (MPCs). MPCs were mock-transduced with lentivirus constitutively expressing only the lenti-rtTA M2 domain without any target gene. The cells were cultured for 7 days. At day 7, MPCs received DMSO. At day 8, cell culture was collected for further analysis.	mesenchymal progenitor cell (ESC)<br>Differentiated mesenchymal progenitor-like cells (MPC) derived from human pluripotent, embryonic stem cells.	3
15	GSE57896	adipocyte conversion study 1 (wac; R406; 24h)	White adipocytes (WAC) differentiated from human pluripotent, embryonic stem cell line (hESC SA001) were treated with R406 for 24 hours. hESCs were differentiated as embryoid bodies and then re-plated and passaged to generate mesenchymal progenitor cells (MPCs). MPCs were transduced (programmed) with lentivirus constitutively expressing the lenti-rtTA M2 domain and virus carrying the inducible cDNA transgenes PPARG2 (lenti-PPARG2). All differentiated cells were cultured in media containing adipogenic factors and doxycycline for 7 days. At day 7, when pluripotent stem cell-derived white adipocytes (PSC-WA) were differentiating, R406 (1 µM) was applied to PSC-WA. At day 8, cell culture was collected for further analysis. R406 is a potent inhibitor of signaling through Spleen Tyrosine Kinase (SYK). ATC code:---	adipocyte - white (ESC)<br>White adipocytes-like cells (WA) differentiated from human pluripotent, embryonic stem cells.	3	mock treated mesenchymal progenitor cell sample (24h)	Differentiated mesenchymal progenitor cells (MPCs) derived from human pluripotent, embryonic stem cell line (hESC SA001) were treated with DMSO for 24 hours. hESCs were differentiated as embryoid bodies and then re-plated and passaged to generate mesenchymal progenitor cells (MPCs). MPCs were mock-transduced with lentivirus constitutively expressing only the lenti-rtTA M2 domain without any target gene. The cells were cultured for 7 days. At day 7, MPCs received DMSO. At day 8, cell culture was collected for further analysis.	mesenchymal progenitor cell (ESC)<br>Differentiated mesenchymal progenitor-like cells (MPC) derived from human pluripotent, embryonic stem cells.	3

Supplementary Table S5.

Experimental conditions promoting PACAP27/38 accumulation.

		Experimental group				Control group			
ID	Repository	Stimulus	Description	Anatomical Part or Cell line or Neoplasm	n	Stimulus	Description	Anatomical Part or Cell line or Neoplasm	n
1	HS-03651	iPSC-derived GABA neuron study 1	GABAergic neuron samples obtained by ATCC-BXS0116 induced pluripotent stem cells' (iPSCs) differentiation. Sendai virus reprogrammed iPSCs were initially gained from bone marrow CD34+ cells of a healthy female donor. More details about the differentiation process could be found in the supplementary data.	GABA neuron (ATCC-BXS0116)	4	ATCC-BXS0116 cell sample (iPSC)	Induced pluripotent stem cell (iPSC) ATCC-BXS0116 samples. Sendai virus reprogrammed iPSC were initially gained from bone marrow CD34+ cells of a healthy female donor.	ATCC-BXS0116	3
2	HS-01636	TCGA pancreatic cancer study 1 (pancreatic head; infiltr. duct carcinoma; stroma)	Tumor stroma tissue samples obtained from pancreatic head of patient with primary infiltrating duct carcinoma.	head of pancreas, infiltrating duct carcinoma, NOS, stroma, ICD-O-3:M8500/3	7	TCGA pancreatic cancer study 1 (pancreatic head; infiltr. duct carcinoma)	Primary tumor tissue samples obtained from the pancreatic head of patients with infiltrating duct carcinoma.	head of pancreas, infiltrating duct carcinoma, NOS, stroma, ICD-O-3:M8500/3	116
3	HS-03651	iPSC-derived sensory neuron study 1	Sensory neuron samples obtained by ATCC-BXS0116 induced pluripotent stem cells' (iPSCs) differentiation. Sendai virus reprogrammed iPSCs were initially gained from bone marrow CD34+ cells of a healthy female donor. More details about the differentiation process could be found in the supplementary data.	sensory neuron (ATCC-BXS0116)	4	ATCC-BXS0116 cell sample (iPSC)	Induced pluripotent stem cell (iPSC) ATCC-BXS0116 samples. Sendai virus reprogrammed iPSC were initially gained from bone marrow CD34+ cells of a healthy female donor.	ATCC-BXS0116	3
4	HS-01637	TCGA pheochromocytoma study 1 (malignant; primary)	Primary tumor tissue samples obtained from the adrenal gland of patients with malignant pheochromocytoma.	adrenal gland, pheochromocytoma, malignant	39	adjacent adrenal gland tissue TCGA	Adjacent histologically normal adrenal gland tissue samples from patients with pheochromocytoma (NOS).	adrenal gland	3
5	HS-03416	focal cortical dysplasia study 1 (IIa)	Cerebral cortex (occipital, frontal or temporal lobe) samples collected from pediatric patients with focal cortical dysplasia (FCD) type IIa ((classified by 2011 International League Against Epilepsy (ILAE)). Histopathologically type IIa was characterized by presence of dysmorphic neurons, which presented with a significantly enlarged cell body and nucleus, malorientation, and cytoplasmic accumulation of neurofilament protein (SMI32). The cohort's mean age was 5.5 ± 1.4 (mean age ± standard error of mean (SEM).	cerebral cortex lobe / region	6	control brain tissue	Cerebral cortex (temporal or frontal lobe) samples collected from control non-epileptic patients. Study inclusion criteria were a) age <25 years; b) for autopsies a postmortem range <24 hours; c) no prior history of seizures; d) and no signs of autolysis upon histopathologic brain examination.	cerebral cortex lobe / region	8
6	HS-01637	TCGA pheochromocytoma study 1 (NOS; primary)	Primary tumor sample obtained from the adrenal gland of patients with pheochromocytoma (NOS).	adrenal gland, pheochromocytoma, NOS	107	adjacent adrenal gland tissue TCGA	Adjacent histologically normal adrenal gland tissue samples from patients with pheochromocytoma (NOS).	adrenal gland	3
7	HS-03664	iPSC-derived sensory neuron study 2 (2m)	Young sensory neurons (2 months) differentiated from induced pluripotent stem cells (iPSC) that were obtained by reprogramming of skin fibroblasts from healthy donors.	sensory neuron (iPSC)	8	normal iPSC cell sample	Human induced pluripotent stem cells derived from dermal fibroblasts of healthy donors.	iPSC (dermal fibroblast)	4
8	HS-03664	iPSC-derived sensory neuron study 2 (6m; L-serine)	L-serine treated (10 mM) old sensory neurons (6 months) differentiated from induced pluripotent stem cells (iPSC) that were obtained by reprogramming of skin fibroblasts from healthy donors.	sensory neuron (iPSC)	4	normal iPSC cell sample	Human induced pluripotent stem cells derived from dermal fibroblasts of healthy donors.	iPSC (dermal fibroblast)	4
9	HS-03664	hereditary sensory neuropathy type 1 study 1 (sensory neuron; 2m)	Young sensory neurons (2 months) differentiated from induced pluripotent stem cells (iPSC) that were obtained by reprogramming of skin fibroblasts from patients with hereditary sensory neuropathy type 1 (HSN1).	sensory neuron (iPSC)	6	hereditary sensory neuropathy type 1 study 1 (iPSC)	Human induced pluripotent stem cells derived from dermal fibroblasts of patients with hereditary sensory neuropathy type 1 (HSN1).	iPSC (dermal fibroblast)	3
10	HS-03664	hereditary sensory neuropathy type 1 study 1 (sensory neuron; 6m)	Old sensory neurons (6 months) differentiated from induced pluripotent stem cells (iPSC) that were obtained by reprogramming of skin fibroblasts from patients with hereditary sensory neuropathy type 1 (HSN1).	sensory neuron (iPSC)	3	hereditary sensory neuropathy type 1 study 1 (iPSC)	Human induced pluripotent stem cells derived from dermal fibroblasts of patients with hereditary sensory neuropathy type 1 (HSN1).	iPSC (dermal fibroblast)	3
11	HS-03664	hereditary sensory neuropathy type 1 study 1 (sensory neuron; 6m; L-serine)	L-serine treated (10 mM) old sensory neurons (6 months) differentiated from induced pluripotent stem cells (iPSC) that were obtained by reprogramming of skin fibroblasts from patients with hereditary sensory neuropathy type 1 (HSN1).	sensory neuron (iPSC)	3	hereditary sensory neuropathy type 1 study 1 (iPSC)	Human induced pluripotent stem cells derived from dermal fibroblasts of patients with hereditary sensory neuropathy type 1 (HSN1).	iPSC (dermal fibroblast)	3
12	HS-03664	iPSC-derived sensory neuron study 2 (6m)	Old sensory neurons (6 months) differentiated from induced pluripotent stem cells (iPSC) that were obtained by reprogramming of skin fibroblasts from healthy donors.	sensory neuron (iPSC)	4	normal iPSC cell sample	Human induced pluripotent stem cells derived from dermal fibroblasts of healthy donors.	iPSC (dermal fibroblast)	4
13	HS-03662	stem cell differentiation study 59 (iDRG; 15d)	Immature dorsal root ganglia neurons (iDRGs) obtained by differentiation of WA09 embryonic stem cells. WA09 cells were differentiated for 8 days and subsequently cryopreserved. After thawing, cells were further differentiated for 7 days. Further details are described in the paper.	iDRG (WA09)	4	stem cell differentiation study 59 (iDRG; 9d)	Immature dorsal root ganglia neurons (iDRGs) obtained by differentiation of WA09 embryonic stem cells. WA09 cells were differentiated for 8 days and subsequently cryopreserved. After thawing, cells were further differentiated for 1 day. Further details are described in the paper.	iDRG (WA09)	4
14	HS-03662	stem cell differentiation study 59 (iDRG; 15d)	Immature dorsal root ganglia neurons (iDRGs) obtained by differentiation of WA09 embryonic stem cells. WA09 cells were differentiated for 8 days and subsequently cryopreserved. After thawing, cells were further differentiated for 7 days. Further details are described in the paper.	iDRG (WA09)	4	stem cell differentiation study 59 (8d)	WA09 embryonic stem cell samples differentiated for 8 days. Further details are described in the paper.	WA09	4

15	HS-00953	psoriasis study 5 (Zaba; lesional; baseline; resp.)	Lesional skin punch biopsies derived from patients with moderate to severe plaque psoriasis (Zaba (Amgen) data set), classified as responders to etanercept treatment.	skin	11	psoriasis study 5 (Zaba; non-lesional; baseline; resp.)	Non-lesional skin punch biopsies derived from patients with moderate to severe plaque psoriasis (Zaba (Amgen) data set), classified as responders to etanercept treatment.	skin	11
16	HS-02935	psoriasis study 24 (lesional; baseline; etarnecept; non-responder)	Lesional skin punch biopsies derived from patients with moderate-to-severe psoriasis at baseline (prior to treatment with etarnecept) and assigned as non-responders. Non-responders are patients who did not achieve ≥75% improvement from baseline in Psoriasis Area and Severity Index (PASI75) at week 12. Lesional skin samples were isolated from a representative psoriatic target lesion (≥ 3cm). Patients participated in a phase 3 of Psoriasis Trial ACCEPT.	skin	13	psoriasis study 24 (non-lesional; baseline; etarnecept; non-responder)	Non-lesional and macroscopic normal skin punch biopsies derived from patients with moderate-to-severe psoriasis at baseline (prior to treatment with etarnecept) and assigned as non-responders. Non-responders are patients who did not achieve ≥75% improvement from baseline in Psoriasis Area and Severity Index (PASI75) at week 12. Patients participated in a phase 3 of Psoriasis Trial ACCEPT.	skin	13
17	HS-00900	psoriasis study 3 (lesional)	Lesional skin punch biopsies derived from patients with moderate-to-severe psoriasis vulgaris who were not receiving active psoriasis therapy. Lesional skin samples were isolated from a representative psoriatic target lesion (≥ 3cm).	skin	83	psoriasis study 3 (non-lesional)	Macroscopic normal, non-lesional skin punch biopsies derived from patients with moderate-to-severe psoriasis vulgaris who were not receiving active psoriasis therapy.	skin	83
18	HS-02936	psoriasis study 25 (lesional; baseline; etarnecept; non-responder)	Lesional skin punch biopsies derived from patients with moderate-to-severe psoriasis at baseline (prior to treatment with etarnecept) and assigned as non-responders. Non-responders are patients who did not achieve ≥75% improvement from baseline in Psoriasis Area and Severity Index (PASI75) at week 12. Lesional skin samples were isolated from a representative psoriatic target lesion (≥ 3cm). Patients participated in a phase 3 of Psoriasis Trial ACCEPT.	skin	14	psoriasis study 25 (non-lesional; baseline; etarnecept; non-responder)	Non-lesional and macroscopic normal skin punch biopsies derived from patients with moderate-to-severe psoriasis at baseline (prior to treatment with etarnecept) and assigned as non-responders. Non-responders are patients who did not achieve ≥75% improvement from baseline in Psoriasis Area and Severity Index (PASI75) at week 12. Patients participated in a phase 3 of Psoriasis Trial ACCEPT.	skin	13
19	HS-03662	stem cell differentiation study 59 (iDRG; 15d)	Immature dorsal root ganglia neurons (iDRGs) obtained by differentiation of WA09 embryonic stem cells. WA09 cells were differentiated for 8 days and subsequently cryopreserved. After thawing, cells were further differentiated for 7 days. Further details are described in the paper.	iDRG (WA09)	4	normal embryonic stem cell sample (WA09)	Undifferentiated WA09 embryonic stem cell samples.	WA09	4
20	HS-00002	expO lung cancer study 1 (neuroendocrine carcinoma; primary)	Primary tumor tissue samples obtained from the lung of patients with neuroendocrine carcinoma.	bronchus or lung, neuroendocrine carcinoma	6	expO lung cancer study 1 (adenocarcinoma, NOS; primary)	Primary tumor tissue samples obtained from the lung of patients with adenocarcinoma (NOS).	bronchus or lung, adenocarcinoma, NOS	36
21	HS-03662	stem cell differentiation study 59 (iDRG; 12d)	Immature dorsal root ganglia neurons (iDRGs) obtained by differentiation of WA09 embryonic stem cells. WA09 cells were differentiated for 8 days and subsequently cryopreserved. After thawing, cells were further differentiated for 4 days. Further details are described in the paper.	iDRG (WA09)	4	stem cell differentiation study 59 (iDRG; 9d)	Immature dorsal root ganglia neurons (iDRGs) obtained by differentiation of WA09 embryonic stem cells. WA09 cells were differentiated for 8 days and subsequently cryopreserved. After thawing, cells were further differentiated for 1 day. Further details are described in the paper.	iDRG (WA09)	4
22	HS-03662	stem cell differentiation study 59 (iDRG; 12d)	Immature dorsal root ganglia neurons (iDRGs) obtained by differentiation of WA09 embryonic stem cells. WA09 cells were differentiated for 8 days and subsequently cryopreserved. After thawing, cells were further differentiated for 4 days. Further details are described in the paper.	iDRG (WA09)	4	stem cell differentiation study 59 (8d)	WA09 embryonic stem cell samples differentiated for 8 days. Further details are described in the paper.	WA09	4
23	HS-01021	ulcerative colitis study 9 (prior therapy)	Colon biopsies derived from pediatric patients diagnosed with ulcerative colitis at time of enrollment. Patients were not receiving medications for the treatment of inflammatory bowel disease at the time the biopsy was taken.	colon	8	normal colon tissue	Colon biopsies derived from pediatric healthy controls.	colon	11
24	HS-00899	ulcerative colitis study 7 (inflamed)	Colon mucosa biopsies derived from patients with active ulcerative colitis (inflamed). Adjacent mucosal colonic biopsies were obtained endoscopically from the descending colon.	colonic mucosa	8	colon mucosa tissue (irritable bowel syndrome)	Colon mucosa biopsies derived from patients with irritable bowel syndrome. Adjacent mucosal colonic biopsies were obtained endoscopically from the descending colon.	colonic mucosa	9
25	HS-03662	stem cell differentiation study 59 (iDRG; 9d)	Immature dorsal root ganglia neurons (iDRGs) obtained by differentiation of WA09 embryonic stem cells. WA09 cells were differentiated for 8 days and subsequently cryopreserved. After thawing, cells were further differentiated for 1 day. Further details are described in the paper.	iDRG (WA09)	4	stem cell differentiation study 59 (8d)	WA09 embryonic stem cell samples differentiated for 8 days. Further details are described in the paper.	WA09	4
26	HS-03662	stem cell differentiation study 59 (iDRG; 12d)	Immature dorsal root ganglia neurons (iDRGs) obtained by differentiation of WA09 embryonic stem cells. WA09 cells were differentiated for 8 days and subsequently cryopreserved. After thawing, cells were further differentiated for 4 days. Further details are described in the paper.	iDRG (WA09)	4	normal embryonic stem cell sample (WA09)	Undifferentiated WA09 embryonic stem cell samples.	WA09	4
27	HS-02624	Merkel cell carcinoma study 3 (primary)	Primary tumor tissue from the skin of patients with Merkel cell carcinoma.	skin, Merkel cell carcinoma	19	normal skin tissue	Normal skin samples from healthy donors.	skin	64
28	HS-02624	Merkel cell carcinoma study 3 (metastatic)	Metastatic tumor tissue from different metastatic sites (skin, lymph node, parotid gland) of patients with Merkel cell carcinoma of the skin.	skin, Merkel cell carcinoma, metastatic	11	normal skin tissue	Normal skin samples from healthy donors.	skin	64
29	HS-03662	stem cell differentiation study 59 (iDRG; 9d)	mmature dorsal root ganglia neurons (iDRGs) obtained by differentiation of WA09 embryonic stem cells. WA09 cells were differentiated for 8 days and subsequently cryopreserved. After thawing, cells were further differentiated for 1 day. Further details are described in the paper.	iDRG (WA09)	4	normal embryonic stem cell sample (WA09)	Undifferentiated WA09 embryonic stem cell samples.	WA09	4

30	HS-03228	ulcerative colitis study 67 (ascending; inflamed)	Intestinal epithelial cells isolated from inflammatory site of ascending colon of treatment-naive paediatric patients with ulcerative colitis at diagnostic endoscopy. The inflammation status was made based on the histology of a paired sample taken within 2 cm of samples. Intestinal epithelial cells were purified with enzyme digestion and extracted using bead sorting for the epithelial cell adhesion molecule (EpCAM) positive cells.	ascending colonic epithelium cell	3	normal ascending colonic epithelium cell sample	Control intestinal epithelial cells isolated from ascending colon of healthy children. Cells were isolated from macroscopically and histologically normal non-inflammatory mucosa, purified with enzyme digestion and extracted using bead sorting for the epithelial cell adhesion molecule (EpCAM) positive cells.	ascending colonic epithelium cell	5
31	HS-01563	small cell lung cancer study 3	Primary tumor tissue samples of patients with small cell lung carcinoma.	bronchus or lung, small cell carcinoma, NOS	74	adjacent lung tissue	Normal lung tissue samples adjacent to tumor from patients with primary small cell lung carcinoma.	lung	7
32	HS-03135	small cell lung cancer study 8 (lung tumor)	Primary tumor tissue samples of patients with small cell lung cancer (SCLC).	bronchus or lung, small cell carcinoma, NOS	31	adjacent lung tissue sample	Normal lung tissue samples adjacent to primary tumor of patients with small cell lung cancer (SCLC).	lung	25

Supplementary Table S6.

Experimental conditions promoting PACAP27/38 depletion.

		Experimental group				Control group			
ID	Repository	Stimulus	Description	Anatomical Part or Cell line or Neoplasm	n	Stimulus	Description	Anatomical Part or Cell line or Neoplasm	n
1	HS-01587	glioma study 20 (tumor stroma	RI-localized biopsies of gliomas (primary or recurrent). Samples measuring ~1.0 cm × 0.5 cm × 0.5 cm were obtained before surgical debulking. Sampled regions included only areas within the gadolinium enhancing core of the tumors. The average age of patient was 61.7 ± 1.9 years (SEM). Female to male ratio was 11:16. Some of the patients were on Temozolomide, Bevacizamab or a radiation therapy. All patients with recurrent glioblastoma (9) had previous surgery for GBM and had received adjuvant therapy (Temozolomide in combination with radiation).	brain, glioma, malignant, NOS	39	normal cerebral cortex tissue	Non-neoplastic brain tissue samples were collected from 11 patients, with no oncological history, who were undergoing ventriculoperitoneal shunt placement for normal pressure hydrocephalus or surgical resection for seizure control. A small biopsy was obtained at the cortical entry point before passing the ventricular catheter.	cerebral cortex (neopallium)	17
2	HS-03654	diabetes type 1 study 24 (MN-iPSC; neurite; normoxia)	T1D iPSC-derived human motor neuron (MN-iPSC) neurite samples. iPSC were derived from patients with type 1 diabetes (T1D). Motor neurons were cultured under normoxic (atmospheric) conditions for 24 hours. The neurite cell compartment was achieved by cell fractioning afterwards.	motor neuron (iPSC)	4	diabetes type 1 study 24 (MN-iPSC; soma; normoxia)	T1D iPSC-derived human motor neuron (MN-iPSC) soma samples. iPSC were derived from patients with type 1 diabetes (T1D). Motor neurons were cultured under normoxic (atmospheric) conditions for 24 hours. The soma cell compartment was achieved by cell fractioning afterwards.	motor neuron (iPSC)	4
3	HS-03654	ESC-derived motor neuron study 1 (neurite)	Human ESC-derived human motor neuron (MN-ESC) neurite samples. Motor neurons were cultured under normoxic (atmospheric) conditions for 24 hours. The neurite cell compartment was achieved by cell fractioning afterwards.	motor neuron (ESC)	3	ESC-derived motor neuron study 1 (soma)	Human ESC-derived human motor neuron (MN-ESC) soma samples. Motor neurons were cultured under normoxic (atmospheric) conditions for 24 hours. The soma cell compartment was achieved by cell fractioning afterwards.	motor neuron (ESC)	3
4	HS-03715	iPSC-derived sensory neuron study 3 (NC-iSN2; short; touch)	Human neural crest-derived induced sensory neurons (NC-iSN2) derived from iPSC (GM25256 (WTC-11)) by stable expression of NGN2-BRN3A under the control of a doxycycline-inducible promoter. Doxycycline was supplemented in medium for 24 hours (short term). Briefly, to generate NC-iSN2 samples, on day -1 iPSCs were dissociated and seeded in a plate for spheroid formation in E8 medium with Y-27632. On day 0, spheroids were transferred to uncoated tissue culture-treated dishes in NDM with different supplements (see publication). This medium was changed every other day. After 1-2 weeks, spheroids spontaneously attached to the culture surface and neural crest cells migrated outward. All spheroids were manually removed, and the neural crest cells were re-plated on polyethylenimine and laminin-coated dishes in NDM – this is denoted as day 0 of neural induction. For NC-iSN2s, doxycycline was added on day 0 for 24 hours (short term). NTFs and 50 nM retinoic acid were supplemented from day 0 onwards. On day 2, the neural crest cells were re-plated for the final time onto polyethylenimine and laminin-coated dishes. Cells were collected for analysis on day 21. Sensory neurons can transduce mechanical stimuli only. Medium was lacking small-molecule inhibitors traditionally used for neural differentiation.	sensory neuron (GM25256 NGN2-BRN3A iPSC)	3	iPSC-derived sensory neuron study 3 (iSN; cold; touch)	Human induced sensory neurons (iSN) derived from iPSC (GM25256 (WTC-11)) by stable expression of NGN2-BRN3A under the control of a doxycycline-inducible promoter. Doxycycline was supplemented in medium for 14 days (long term). Briefly, to generate iSN samples, on day 0 stable transfected iPSCs were dissociated and plated on Matrigel-coated dishes in neural differentiation medium (NDM) supplemented with Y-27632 and doxycycline. On day 2, the cells were dissociated and were seeded on plates coated with polyethylenimine and laminin in NDM with doxycycline. Starting on day 8 onward, neurotrophic factors were added to the NDM. Doxycycline was discontinued starting on day 14. Cells were collected for analysis on day 21. Sensory neurons can transduce both cold and mechanical stimuli. Medium was lacking small-molecule inhibitors traditionally used for neural differentiation.	sensory neuron (GM25256 NGN2-BRN3A iPSC)	3
5	HS-01587	glioma study 20 (tumor margin)	MRI-localized biopsies of gliomas (primary or recurrent). Samples measuring ~1.0 cm × 0.5 cm × 0.5 cm were obtained before surgical debulking. Sampled regions included only areas of nonenhancing, FLAIR hyperintense tissue at the margins of the tumors. The average age of patient was 61.7 ± 1.9 years (SEM). Female to male ratio was 11:16. Some of the patients were on Temozolomide, Bevacizamab or a radiation therapy. All patients with recurrent glioblastoma (9) had previous surgery for GBM and had received adjuvant therapy (Temozolomide in combination with radiation).	brain, glioma, malignant, NOS	36	normal cerebral cortex tissue	Non-neoplastic brain tissue samples were collected from 11 patients, with no oncological history, who were undergoing ventriculoperitoneal shunt placement for normal pressure hydrocephalus or surgical resection for seizure control. A small biopsy was obtained at the cortical entry point before passing the ventricular catheter.	cerebral cortex (neopallium)	17
6	HS-01618	TCGA brain cancer study 1 (brain; glioblastoma, NOS; relapse)	Relapse tumor tissue samples from the brain of patients with glioblastoma (NOS).	brain, glioblastoma, NOS	13	adjacent brain tumor tissue TCGA	Normal adjacent brain tissue samples derived from patients with brain cancer.	brain (encephalon)	5
7	HS-03639	Fel d1 IgE (18h); Fel d 1 (4h) study 1	Bone marrow derived mast cells primed with Fel d 1-specific IgE (10nM) for 18 hours, and activated with Fel d 1 (1nM) antigen for 4h. Bone marrow-CD133+ cells of healthy donors were cultured in StemSpan SFEM media in the presence of 50ng/mL IL-6, 100ng/mL SCF and 1ng/mL IL-3 for 3 weeks and then with IL-6 and SCF only for 4 more weeks. Generated mast cells were defined as CD117+/FceRIa+ cells based on cell surface marker expression by flow cytometry.	bone marrow derived mast cell	4	untreated bone marrow derived mast cell sample	Untreated bone marrow derived mast cells. Bone marrow-CD133+ cells of healthy donors were cultured in StemSpan SFEM media in the presence of 50ng/mL IL-6, 100ng/mL SCF and 1ng/mL IL-3 for 3 weeks and then with IL-6 and SCF only for 4 more weeks. Generated mast cells were defined as CD117+/FceRIa+ cells based on cell surface marker expression by flow cytometry.	bone marrow derived mast cell	4

8	HS-01618	TCGA brain cancer study 1 (brain; glioblastoma, NOS; primary)	Primary tumor tissue samples from the brain of patients with glioblastoma (NOS).	brain, glioblastoma, NOS	155	adjacent brain tumor tissue TCGA	Normal adjacent brain tissue samples derived from patients with brain cancer.	brain (encephalon)	5
9	HS-03639	IL-13 (24h); Fel d 1 IgE (18h); Fel d 1 (4h) study 1	Bone marrow derived mast cells treated with interleukin 13 (IL-13, 100nM) for 24 hours, primed with Fel d 1-specific IgE (10nM) for 18 hours, and activated with Fel d 1 antigen (1nM) for 4h. Bone marrow-CD133+ cells of healthy donors were cultured in StemSpan SFEM media in the presence of 50ng/mL IL-6, 100ng/mL SCF and 1ng/mL IL-3 for 3 weeks and then with IL-6 and SCF only for 4 more weeks. Generated mast cells were defined as CD117+/FceRIa+ cells based on cell surface marker expression by flow cytometry.	bone marrow derived mast cell	4	untreated bone marrow derived mast cell sample	Untreated bone marrow derived mast cells. Bone marrow-CD133+ cells of healthy donors were cultured in StemSpan SFEM media in the presence of 50ng/mL IL-6, 100ng/mL SCF and 1ng/mL IL-3 for 3 weeks and then with IL-6 and SCF only for 4 more weeks. Generated mast cells were defined as CD117+/FceRIa+ cells based on cell surface marker expression by flow cytometry.	bone marrow derived mast cell	4
10	HS-01618	TCGA brain cancer study 1 (cerebrum; astrocytoma, anaplastic type; primary)	Primary tumor tissue samples from the cerebrum of patients with astrocytoma (anaplastic type).	cerebrum, astrocytoma, anaplastic type	107	adjacent brain tumor tissue TCGA	Normal adjacent brain tissue samples derived from patients with brain cancer.	brain (encephalon)	5
11	HS-03639	IL-4 (24h); Fel d 1 IgE (18h); Fel d 1 (4h) study 1	Bone marrow derived mast cells treated with interleukin 4 (IL-4, 100nM) for 24 hours, primed with Fel d 1-specific IgE (10nM) for 18 hours, and activated with Fel d 1 antigen (1nM) for 4h. Bone marrow-CD133+ cells of healthy donors were cultured in StemSpan SFEM media in the presence of 50ng/mL IL-6, 100ng/mL SCF and 1ng/mL IL-3 for 3 weeks and then with IL-6 and SCF only for 4 more weeks. Generated mast cells were defined as CD117+/FceRIa+ cells based on cell surface marker expression by flow cytometry.	bone marrow derived mast cell	4	IL-4 study 9	Bone marrow derived mast cells treated with interleukin 4 (IL-4, 100nM) for 24 hours. Bone marrow-CD133+ cells of healthy donors were cultured in StemSpan SFEM media in the presence of 50ng/mL IL-6, 100ng/mL SCF and 1ng/mL IL-3 for 3 weeks and then with IL-6 and SCF only for 4 more weeks. Generated mast cells were defined as CD117+/FceRIa+ cells based on cell surface marker expression by flow cytometry.	bone marrow derived mast cell	4
12	HS-03639	IL-13 (24h); Fel d 1 IgE (18h); Fel d 1 (4h) study 1	Bone marrow derived mast cells treated with interleukin 13 (IL-13, 100nM) for 24 hours, primed with Fel d 1-specific IgE (10nM) for 18 hours, and activated with Fel d 1 antigen (1nM) for 4h. Bone marrow-CD133+ cells of healthy donors were cultured in StemSpan SFEM media in the presence of 50ng/mL IL-6, 100ng/mL SCF and 1ng/mL IL-3 for 3 weeks and then with IL-6 and SCF only for 4 more weeks. Generated mast cells were defined as CD117+/FceRIa+ cells based on cell surface marker expression by flow cytometry.	bone marrow derived mast cell	4	IL-13 study 4	Bone marrow derived mast cells treated with interleukin 13 (IL-13, 100nM) for 24 hours. Bone marrow-CD133+ cells of healthy donors were cultured in StemSpan SFEM media in the presence of 50ng/mL IL-6, 100ng/mL SCF and 1ng/mL IL-3 for 3 weeks and then with IL-6 and SCF only for 4 more weeks. Generated mast cells were defined as CD117+/FceRIa+ cells based on cell surface marker expression by flow cytometry.	bone marrow derived mast cell	4
13	HS-01582	autism study 11	Cerebral cortical tissue obtained postmortem from subjects with autism.	Brodmann area 9 anterior transverse temporal area (Brodmann area 41) posterior superior temporal gyrus (Brodmann area 22)	3	normal cerebral cortex tissue	Cerebral cortex tissue obtained postmortem from objects without any known neurological disorder.	Brodmann area 9 anterior transverse temporal area (Brodmann area 41)	3
14	HS-01618	TCGA brain cancer study 1 (cerebrum; mixed glioma; relapse)	Relapse tumor tissue samples from the cerebrum of patients with mixed glioma.	cerebrum, mixed glioma	3	adjacent brain tumor tissue TCGA	Normal adjacent brain tissue samples derived from patients with brain cancer.	brain (encephalon)	5
15	HS-01618	TCGA brain cancer study 1 (cerebrum; astrocytoma, NOS; primary)	Primary tumor tissue samples from the cerebrum of patients with astrocytoma (NOS).	cerebrum, astrocytoma, NOS	56	adjacent brain tumor tissue TCGA	Normal adjacent brain tissue samples derived from patients with brain cancer.	brain (encephalon)	5
16	HS-01618	TCGA brain cancer study 1 (brain; astrocytoma, anaplastic type; primary)	Primary tumor tissue samples from the brain of patients with astrocytoma (anaplastic type).	brain, astrocytoma, anaplastic type	19	adjacent brain tumor tissue TCGA	Normal adjacent brain tissue samples derived from patients with brain cancer.	brain (encephalon)	5
17	HS-01617	TCGA bladder cancer study 1 (papillary transitional cell carcinoma)	Primary tumor tissue samples obtained from the urinary bladder of patients with papillary transitional cell carcinoma (papillary urothelial carcinoma).	bladder, papillary transitional cell carcinoma	64	adjacent bladder tissue TCGA	Histological normal lung tissue samples from patients with bladder cancer.	urinary bladder	19
18	HS-01618	TCGA brain cancer study 1 (brain; oligodendroglioma, NOS; primary)	Primary tumor tissue samples from the brain of patients with oligodendroglioma (NOS).	brain, oligodendroglioma, NOS	15	adjacent brain tumor tissue TCGA	Normal adjacent brain tissue samples derived from patients with brain cancer.	brain (encephalon)	5
19	HS-01618	TCGA brain cancer study 1 (cerebrum; mixed glioma; primary)	Primary tumor tissue samples from the cerebrum of patients with mixed glioma.	cerebrum, mixed glioma	110	adjacent brain tumor tissue TCGA	Normal adjacent brain tissue samples derived from patients with brain cancer.	brain (encephalon)	5
20	HS-01618	TCGA brain cancer study 1 (cerebrum; oligodendroglioma, NOS; primary)	Primary tumor tissue samples from the cerebrum of patients with oligodendroglioma (NOS).	cerebrum, oligodendroglioma, NOS	95	adjacent brain tumor tissue TCGA	Normal adjacent brain tissue samples derived from patients with brain cancer.	brain (encephalon)	5
21	HS-01618	TCGA brain cancer study 1 (cerebrum; oligodendroglioma, anaplastic type; primary)	Primary tumor tissue samples from the cerebrum of patients with oligodendroglioma (anaplastic type).	cerebrum, oligodendroglioma, anaplastic type	69	adjacent brain tumor tissue TCGA	Normal adjacent brain tissue samples derived from patients with brain cancer.	brain (encephalon)	5
22	HS-01618	TCGA brain cancer study 1 (brain; mixed glioma; primary)	Primary tumor tissue samples from the brain of patients with mixed glioma.	brain, mixed glioma	12	adjacent brain tumor tissue TCGA	Normal adjacent brain tissue samples derived from patients with brain cancer.	brain (encephalon)	5

23	HS-01618	TCGA brain cancer study 1 (temporal lobe; mixed glioma; primary)	Primary tumor tissue samples from the temporal lobe of patients with mixed glioma.	temporal lobe, mixed glioma	4	adjacent brain tumor tissue TCGA	Normal adjacent brain tissue samples derived from patients with brain cancer.	brain (encephalon)	5
24	HS-01618	TCGA brain cancer study 1 (brain; oligodendroglioma, anaplastic type; primary)	Primary tumor tissue samples from the brain of patients with oligodendroglioma (anaplastic type).	brain, oligodendroglioma, anaplastic type	8	adjacent brain tumor tissue TCGA	Normal adjacent brain tissue samples derived from patients with brain cancer.	brain (encephalon)	5
25	HS-01618	TCGA brain cancer study 1 (cerebrum; oligodendroglioma, NOS; relapse)	Relapse tumor tissue samples from the cerebrum of patients with oligodendroglioma (NOS).	cerebrum, oligodendroglioma, NOS	5	adjacent brain tumor tissue TCGA	Normal adjacent brain tissue samples derived from patients with brain cancer.	brain (encephalon)	5
26	HS-01618	TCGA brain cancer study 1 (brain; astrocytoma, NOS; primary)	Primary tumor tissue samples from the brain of patients with astrocytoma (NOS).	brain, astrocytoma, NOS	7	adjacent brain tumor tissue TCGA	Normal adjacent brain tissue samples derived from patients with brain cancer.	brain (encephalon)	5
27	HS-01618	TCGA brain cancer study 1 (cerebrum; oligodendroglioma, anaplastic type; relapse)	Relapse tumor tissue samples from the cerebrum of patients with oligodendroglioma (anaplastic type).	cerebrum, oligodendroglioma, anaplastic type	4	adjacent brain tumor tissue TCGA	Normal adjacent brain tissue samples derived from patients with brain cancer.	brain (encephalon)	5
28	HS-02642	atopic dermatitis study 12 (lesional; adults)	Lesional skin biopsy samples from adult patients (age range 18-73 years) with long-standing atopic dermatitis.	skin	20	atopic dermatitis study 12 (lesional; children)	Lesional popliteal skin biopsy samples from pediatric patients (age range 3 months-5 years) with early-onset atopic dermatitis. All biopsy specimens were from chronic lesions present for more than 72 hours. All patients had moderate-to-severe disease with recent-onset (within the previous 6 months). Systemic immunosuppressants within the past 4 weeks, topical steroids or immunomodulators within 1 week, or moisturizers within 12 hours before evaluation were restricted. Patients with active skin infections were excluded.	skin	18
29	HS-01016	brain tumor study 1 (ependymoma)	Primary tumor tissue sample from the brain of patients with ependymoma.	brain, ependymoma, NOS	46	normal brain tissue	Histologically normal and non-neoplastic tissue sample from different brain anatomical sites of patients with primary brain tumors.	cerebellum frontal lobe medulla oblongata occipital lobe parietal lobe temporal lobe thalamus (dorsal thalamus)	12
30	HS-00041	Alzheimer's disease study 10	Cortical layer III pyramidal neurons of the superior frontal gyrus of clinically and neuropathologically classified late-onset Alzheimer´s disease afflicted individuals. Subjects in this group had a Braak stage of V or VI with a CERAD score of moderate or frequent.	superior frontal gyrus pyramidal neuron	22	normal pyramidal neuron (superior frontal gyrus)	Cortical layer III pyramidal neurons of the superior frontal gyrus of clinically classified as neurologically normal individuals. Subjects in this group had a Braak stage ranging from I to II with a infrequent Consortium to Establish a Registry for Alzheimer's Disease (CERAD) neuritic plaque density.	superior frontal gyrus pyramidal neuron	9
31	HS-02062	pancreatic islet study 3 (expanded; NIH)	Pancreatic islet cells were expanded according to National Institutes of Health (NIH) protocol for 10 weeks. Expansion phase: 2,000 islet equivalents enriched by retention on a 40-µm filter were seeded onto tissue culture–treated dishes in CMRL-1066 medium containing 2 mmol/l l-glutamine and 10% fetal bovine serum.	pancreatic islet cell	3	normal pancreatic islet sample	Pancreatic islets were obtained from seven donors aged between 37 and 70 years and body mass index between 22 and 27. Functional islets were cultured in CMRL 1066 supplemented with 10% FCS, 1% glutamine, 5.6 mM glucose, 1 mM HEPES, 110 U/ml penicillin, and 110 g/ml streptomycin for 7 days or immediately processed after isolation.	pancreatic islet cell	7
32	HS-02062	pancreatic islet study 3 (re-differentiated; NIH)	Pancreatic islet cells were expanded for 10 weeks and re-differentiated for 1 week according to National Institutes of Health (NIH) protocol. Re-differentiation phase: Expanded cells were cultured for 1 week in serum-free CMRL-1066 medium supplemented with insulin (10 g/ml), transferrin (5.5 g/ml), sodium selenite (6.7ng/ml).	pancreatic islet cell	4	normal pancreatic islet sample	Pancreatic islets were obtained from seven donors aged between 37 and 70 years and body mass index between 22 and 27. Functional islets were cultured in CMRL 1066 supplemented with 10% FCS, 1% glutamine, 5.6 mM glucose, 1 mM HEPES, 110 U/ml penicillin, and 110 g/ml streptomycin for 7 days or immediately processed after isolation.	pancreatic islet cell	7
33	HS-00612	ovarian tumor study 28 (serous cystadenocarcinoma)	Primary tumor tissue sample obtained from the ovary of female patients with malignant serous cystadenocarcinoma.	ovary, serous cystadenocarcinoma, NOS	71	ovarian tumor study 28 (endometrioid carcinoma)	Primary tumor tissue sample obtained from the ovary of female patients with endometrioid carcinoma.	ovary, endometrioid carcinoma	6
34	HS-02062	pancreatic islet study 3 (expanded; Whittier; HGF)	Human pancreatic islets cells were expanded according to Whittier protocol and treated with hepatocyte growth factor (HGF, 25 ng/ml) for 4 weeks. Expansion phase: 1000 islets of 50–150µm in diameter were purified by hand-picking after dithizone staining, partially dissociated using Versene to separate "outer" and "inner" populations, the outer population was removed. Cell clusters from the inner population were plated on HTB-9 matrix-coated dishes in RPMI-1640 supplemented with 2 mM L-glutamine, 10% FBS, and 25 ng/ml HGF. After confluence, cells were harvested using Versene containing 0.025% trypsin and subcultured (1:2).	pancreatic islet cell	3	normal pancreatic islet sample	Pancreatic islets were obtained from seven donors aged between 37 and 70 years and body mass index between 22 and 27. Functional islets were cultured in CMRL 1066 supplemented with 10% FCS, 1% glutamine, 5.6 mM glucose, 1 mM HEPES, 110 U/ml penicillin, and 110 g/ml streptomycin for 7 days or immediately processed after isolation.	pancreatic islet cell	7

35	HS-01016	brain tumor study 1 (glioblastoma)	Primary tumor tissue sample from the brain of patients with glioblastoma multiformae.	brain, glioblastoma, NOS	34	normal brain tissue	Histologically normal and non-neoplastic tissue sample from different brain anatomical sites of patients with primary brain tumors.	cerebellum frontal lobe medulla oblongata occipital lobe parietal lobe temporal lobe thalamus (dorsal thalamus)	12
36	HS-00041	Alzheimer's disease study 9	Cortical layer III pyramidal neurons of the posterior cingulate of clinically and neuropathologically classified late-onset Alzheimer´s disease afflicted individuals. Subjects in this group had a Braak stage of V or VI with a CERAD score of moderate or frequent.	posterior cingulate gyrus pyramidal neuron	9	normal pyramidal neuron (posterior cingulate)	Cortical layer III pyramidal neurons of the posterior cingulate of clinically classified as neurologically normal individuals. Subjects in this group had a Braak stage ranging from I to II with a infrequent Consortium to Establish a Registry for Alzheimer's Disease (CERAD) neuritic plaque density.	posterior cingulate gyrus pyramidal neuron	13
37	HS-01016	brain tumor study 1 (pilocytic astrocytoma)	Primary tumor tissue sample from the brain of patients with pilocytic astrocytoma.	brain, pilocytic astrocytoma	15	normal brain tissue	Histologically normal and non-neoplastic tissue sample from different brain anatomical sites of patients with primary brain tumors.	cerebellum frontal lobe medulla oblongata occipital lobe parietal lobe temporal lobe thalamus (dorsal thalamus)	12
38	HS-01867	colorectal cancer study 30	Formalin-fixed-paraffin-embedded (FFPE) colorectal tissue samples from patients with colorectal cancer.	colon, neoplasm, malignant	45	normal colorectal tissue	Healthy, non-tumor colorectal tissue.	colorectum	4
39	HS-00925	glioma study 5	Primary tumor tissue sample from the supratentorial brain of pediatric patients with glioblastoma multiformae.	brain, glioblastoma, NOS	12	non-tumor brain tissue	Histologically normal brain tissue at rapid autopsy from patients who died from atypical teratoid/rhabdoid tumor.	rontal lobe occipital lobe parietal lobe temporal lobe brain (encephalon) cerebellum	9
40	HS-00041	Alzheimer's disease study 8	Cortical layer III pyramidal neurons of the middle temporal gyrus of clinically and neuropathologically classified late-onset Alzheimer´s disease afflicted individuals. Subjects in this group had a Braak stage of V or VI with a CERAD score of moderate or frequent.	middle temporal gyrus pyramidal neuron	15	normal pyramidal neuron (middle temporal gyrus)	Cortical layer III pyramidal neurons of the middle temporal gyrus of clinically classified as neurologically normal individuals. Subjects in this group had a Braak stage ranging from I to II with a infrequent Consortium to Establish a Registry for Alzheimer's Disease (CERAD) neuritic plaque density.	middle temporal gyrus pyramidal neuron	12
41	HS-00925	atypical teratoid/rhabdoid tumor study 1	Primary tumor tissue sample from the brain of pediatric patients with atypical teratoid/rhabdoid tumor (AT/RT).	brain, atypical teratoid/rhabdoid tumor	20	non-tumor brain tissue	Histologically normal brain tissue at rapid autopsy from patients who died from atypical teratoid/rhabdoid tumor.	cerebellum frontal lobe occipital lobe parietal lobe temporal lobe brain (encephalon)	9
42	HS-00925	primitive neuroectodermal tumor study 1	Primary tumor tissue sample from the supratentorial brain of pediatric patients with primitive neuroectodermal tumor.	brain, primitive neuroectodermal tumor	9	non-tumor brain tissue	Histologically normal brain tissue at rapid autopsy from patients who died from atypical teratoid/rhabdoid tumor.	brain (encephalon) cerebellum frontal lobe occipital lobe parietal lobe temporal lobe	9
